# Magnetoelectric Interactions in Lead-Based and Lead-Free Composites

**DOI:** 10.3390/ma4040651

**Published:** 2011-04-06

**Authors:** Mirza Bichurin, Vladimir Petrov, Anatoly Zakharov, Denis Kovalenko, Su Chul Yang, Deepam Maurya, Vishwas Bedekar, Shashank Priya

**Affiliations:** 1 Institute of Electronic & Information System, Novgorod State University, 173003 Veliky Novgorod, Russia; E-Mails: vladimir.petrov@novsu.ru (V.P.); anatoly.zakharov@novsu.ru (A.Z.); denis.kovalenko@novsu.ru (D.K.); 2 Center for Energy Harvesting Materials and Systems, Virginia Tech, Blacksburg, VA 24061, USA; E-Mails: ysc2573@gmail.com (S.C.Y.); mauryad@vt.edu (D.M.); vishwas@vt.edu (V.B.); spriya@vt.edu (S.P.)

**Keywords:** multiferroics, lead-free piezoelectrics, morphotropic phase boundary, magnetoelectric effect

## Abstract

Magnetoelectric (ME) composites that simultaneously exhibit ferroelectricity and ferromagnetism have recently gained significant attention as evident by the increasing number of publications. These research activities are direct results of the fact that multiferroic magnetoelectrics offer significant technological promise for multiple devices. Appropriate choice of phases with co-firing capability, magnetostriction and piezoelectric coefficient, such as Ni-PZT and NZFO-PZT, has resulted in fabrication of prototype components that promise transition. In this manuscript, we report the properties of Ni-PZT and NZFO-PZT composites in terms of ME voltage coefficients as a function of frequency and magnetic DC bias. In order to overcome the problem of toxicity of lead, we have conducted experiments with Pb-free piezoelectric compositions. Results are presented on the magnetoelectric performance of Ni-NKN, Ni-NBTBT and NZFO-NKN, NZFO-NBTBT systems illustrating their importance as an environmentally friendly alternative.

## 1. Introduction

The realization of a material with simultaneous presence of strong electric and magnetic order at room temperature, termed ‘multiferroics’, would be a milestone for modern electronics and multifunctional materials. Multiferroic magnetoelectric (ME) materials become magnetized when placed in an electric field, and conversely electrically polarized when placed in a magnetic field. Dielectric polarization of a material under a magnetic field, or an induced magnetization under an electric field, requires the simultaneous presence of long-range ordering of magnetic moments and electric dipoles [1,2,3,4]. These materials offer potential for new generations of sensor, filter, and field-tunable microwave dielectric devices [5].

A suitable combination of two phases can yield the magnetoelectric property, such as combination of piezomagnetic and piezoelectric phases or combination of magnetostrictive and piezoelectric phases which will give rise to ME effect. ME effect can also realized by coupled thermal interaction in pyroelectric–pyromagnetic composites. The ME effect obtained in composites is more than one hundred times that of single–phase ME material such as Cr_2_O_3_. Suchtelen and Boomgaard outlined the conceptual points inherent to the ME effect in composites [6,7]. These can be summarized as: (i) Two individual phases should be in equilibrium (ii); Mismatching between grains should not be present (iii); Magnitude of the magnetostriction coefficient of piezomagnetic or magnetostrictive phase and magnitude of the piezoelectric coefficient of the piezoelectric phase must be greater (iv); The accumulated charge must not leak through the piezomagnetic or magnetostrictive phase; and (v) Deterministic strategy for poling of the composites. In spite of the promise of large magnetoelectric (ME) coefficients in elastically coupled nano-composites, experimental investigations for a number of configurations have not yielded values approaching those predicted by continuum mechanics and *ab-initio* calculations. The understanding of the physical interaction occurring in the composites with multi-dimensional connectivity between the magneto-elastic stresses and elastoelectric fields has not been achieved. The lack of this understanding has limited the ability to achieve the theoretical response of the material by coordinating the local electro-magnetic couplings, via coherent elastic interactions between phases. In these materials the theory predicts the size of the ME coefficient to be more than 5 V/cm.Oe. In order to understand the phenomenon of magnetoelectrics in composites comprised of individual piezoelectric and magnetostrictive phases, it will be important to develop theory and experiments that identify the effect of various physical and mechanical parameters on the magnitude of the magnetoelastic and elastoelectric coupling.

The discrepancy between theoretical and experimental values of ME voltage coefficients can also be attributed to the use of a one-dimensional approach. A series of studies by the authors have attempted to address these issues in their models. The suggested method consists of deriving the effective material parameters of composites and is carried out in two stages. In the first stage, the composite is considered as a structure consisting of piezoelectric and magnetostrictive phases. Further, we consider only (symmetric) extensional deformation in this model and ignore any (asymmetric) flexural deformations of the components that would lead to position dependent elastic constants and the need for perturbation procedures. For the polarized piezoelectric phase with the symmetry ¥m, the following equations can be written for the strain and electric displacement:

*^p^S_i_ = ^p^s_ij _^p^T_j_ + ^p^d_ki _^p^E_k_* *^p^D_k_ = ^p^d_ki _^p^T_i_ + ^p^ε_kn _^p^E_n_* (1)

where *^p^S_i_* and *^p^T_j _*are strain and stress tensor components of the piezoelectric phase, *^p^E_k_* and *^p^D_k_* are the vector components of the electric field and electric displacement, *^p^s_ij_* and *^p^d_ki_* are compliance and piezoelectric coefficients, and *^p^ε_kn_* is the permittivity matrix. The magnetostrictive phase is assumed to have a cubic symmetry and is described by the equations:

*^m^S_i_ = ^m^s_ij _^m^T_j_ + ^m^q_ki _^m^H_k_* *^m^B_k_ = ^m^q_ki _^m^T_i_ + ^m^μ_kn _^m^H_n_* (2)

where *^m^S_i_* and *^m^T_j_* are strain and stress tensor components of the magnetostrictive phase, *^m^H_k_* and *^m^B_k_* are the vector components of magnetic field and magnetic induction, *^m^s_ij_* and *^m^q_ki_* are compliance and piezomagnetic coefficients, and *^m^μ_kn_* is the permeability matrix. Equation (2) may be considered as a linearized equation describing the effect of magnetostriction. Using appropriate boundary conditions, the ME voltage coefficients can be obtained by solving Equations (1) and (2). 

In the second stage, the composite is considered to be homogeneous and the behavior is described by:

*S_i_ = s_ij_T_j_ + d_ki_E_k _+ q_ki_H_k_* *D_k_ = d_ki_T_i_ + ε_kn_E_n_* + *α_kn _H_n_* *B_k_ = q_ki_T_i_ +*
*α_kn_E_n_ + μ_kn_H_n_* (3)

where *S_i _*and *T_j _* are strain and stress tensor components, *E_k _*, *D_k_*, *H_k_*, and *B_k_* are the vector components of the electric field, electric displacement, magnetic field and magnetic induction, *s_ij_*,* d_ki_*, and* q_ki_* are effective compliance, piezoelectric and piezomagnetic coefficients, and *ε_kn_*,* μ_kn_* and *α_kn_* are effective permittivity, permeability and ME coefficient. Effective parameters of the composite are obtained by solving Equation (3) by taking into account solutions of Equations (1) and (2). The mechanical strain and stress for composite and homogeneous material are assumed to be the same and the electric and magnetic vectors are determined by using appropriate boundary conditions. This method has shown considerable success in modeling the response of ME composites.

As an example we illustrate the results obtained on L-T mode ME composites. This case corresponds to polarization field *E_0_* and AC electric field *E* perpendicular to the sample plane and bias field *H_0_* and AC magnetic field *H* parallel to the sample plane and the ME coefficient is given as *α_E,T_ =* *α_E,31_* = *E_3_/H_1_*. In this case, non-zero components of *^p^s_ij_*, *^p^d_ki_*, *^m^s_ij _*, *^m^q_ki_, s_ij_*,* d_ki_*, *q_ki_*, α*_kn_* are determined by composite symmetry and Equations (1)–(3) are solved for appropriate boundary conditions. The expression obtained for transverse ME voltage coefficient is given below [8]:

(4) αE,31=E3H1=−v(1−v)(q11m+q21m)pd31ε33p(s12m+s11m)v+ε33p(s11p+s12p)(1−v)−2d312p(1−v)

where *v* is piezoelectric volume fraction. Equation (4) describes the dependence of ME parameters on volume fraction and is used to estimate the ME coupling for some representative systems. For a bilayer of nickel ferrite (NFO) and PZT the measured and calculated values of ME voltage coefficient equal about 240 mV/cm.Oe.

Investigations have revealed the presence of both ferroelectricity and magnetism in a number of materials such as perovskite type BiFeO_3_, BiMnO_3_, the boracite family, BaMF_4_ compounds (M, divalent transition metal ions), hexagonal RMnO_3_ (R, rare earths), and the rare earth molybdates, but none seem to provide large coupling between them [6,7,9,10,11,12,13,14,15,16,17]. Recently, rare earth manganites such as TbMnO_3_, DyMnO_3_, and TbMn_2_O_5_ have been reported to exhibit reproducible electric polarization under magnetic fields, however the magnitude of the magnetoelectric (ME) coefficient (unit of V/cm.Oe) is quite small [4,11,18,19,20,21,22,23]. Single phase materials suffer from the drawback that the ME effect is considerably weak even at low temperatures. Better alternatives to single phase materials are ME composites. The composites exploit the product property of the materials (the ME effect is not present in individual phases). Recently, the focus of much research has been on the laminated magnetoelectric composites made by using piezoelectric and magnetostrictive materials [24,25,26,27,28,29,30,31,32,33,34,35,36,37,38,39,40,41,42,43]. In our original work, we reported the results on laminate composites (MLCs) made from the giant magnetostrictive material, Terfenol-D, and relaxor-based piezocrystals Pb(Mg_1/3_Nb_2/3_)O_3_–PbTiO_3_ (PMN-PT) [44]. Magnetoelectric behavior in laminate composites has now been reported for various material couples including Pb(Zr,Ti)O_3_ (PZT) or Pb(Mg_1/3_Nb_2/3_O_3_-PbTiO_3_ (PMN-PT) layers laminated with magnetostrictive Tb_1−x_Dy_x_Fe_2−y_, Permendur, Ni_1−x_Co_x_Fe_2_O_4_ (*i.e*., NFO), or Co_1−x_Zn_x_Fe_2_O_4_ (*i.e*., CFO) ones [45,46,47,48,49,50,51,52,53,54,55,56,57,58,59,60]. Recently, Bichurin *et al*. found a giant enhancement in the magnitude of the bulk composites at the resonance frequency of the samples [61]. A high ME coefficient of 23000 mV/cm.Oe was reported for the samples with 80% PZT-20% Ni Ferrite (~350 kHz, sample diameter 10 mm), an increase by a factor of 600 as compared to the data at low frequency (~1 kHz).

**Figure 1:**
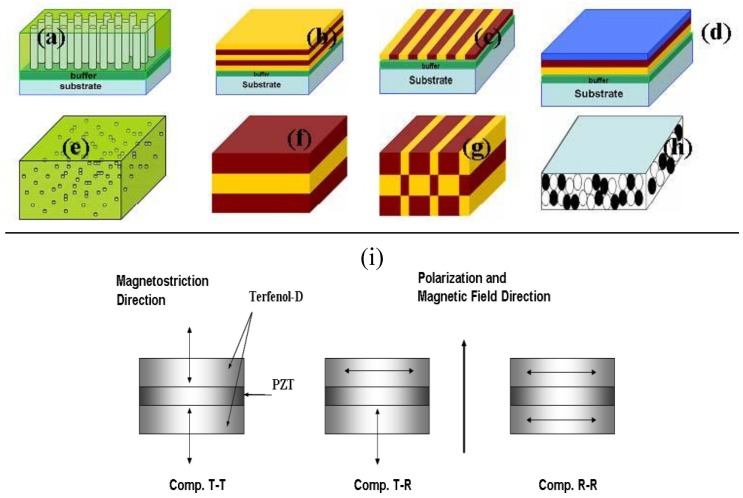
Schematics of the magnetoelectric composites illustrating the multiple possibilities **(a)** self-assembled nano-pillar structures; **(b)** layer by layer deposition; **(c)** deposition accompanied by masking and patterning; **(d)** three phase deposition for gradient materials; **(e)** particles dispersed in a polymer matrix; **(f)** lamination; **(g)** checkerboard arrangement; **(h)** sintered particulates; and **(i)** variation in poling, applied field direction and measuring electrodes.

Figure 1 shows some examples of the structures that can be designed using the two phases in thin film and bulk forms. It can be seen from this figure that the possibilities are numerous and there can be several phases (amorphous, ceramic, metal, polymer, *etc*.), shapes (disk, cylinder, plate, toroid, sphere, *etc*.), and sizes (number of layers, layer thicknesses, length and width can be varied differently) for obtaining the magnetoelectric properties. It is important to note here that all of these combinations will exhibit ME response with varying degree of magnitude. For n phases the number of connectivity patterns is given as (n+3)!/3!n! , which for two phase composites comes out to be 10, for three phases as 20, and 35 for four phase pattern. Further, in each of these shapes there is the possibility of orienting the polarization along different axes and applying the electric (E) and magnetic (H) fields along different axes. In addition there are several choices for materials depending on magnetostriction constant, resistivity, permeability, permittivity, piezoelectric strain and voltage constant, sintering temperature, and chemical reactivity (Magnetostrictive-MnFe_2_O_4_, CoFe_2_O_4_, NiFe_2_O_4_, ZnFe_2_O_4_, YFe_5_O_12_, SmFe_5_O_12_, YIG, Terfenol-D, Metglas 2605SC, Ni, Co, *etc*.; Piezoelectric-PZT, BaTiO_3_, PMN-PT, PVDF, SrBi_4_Ti_4_O_15_, (Na_0.5_K_0.5_)NbO_3_) *etc*.).

Pb(Zr,Ti)O_3_ (PZT) based ceramics have excellent dielectric and piezoelectric properties and are currently the dominant material system for actuators, sensors and resonators. However, the Pb element in these materials presents an environmental problem. Thus, the research is now focused on finding an alternative for PZT’s. In the past two decades, several publications and patents have reported on lead-free piezoelectrics. Out of all the possible choices, (Na,K)NbO_3_ (NKN) based ceramics (e.g., solid solution of NKN-LiNbO_3_ [62], NKN-LiTaO_3_ [63], NKN-LiSbO_3_ [64], NKN-Li(Nb, Ta, Sb)O_3_ [65], NKN-BaTiO_3_ [66], NKN-SrTiO_3_ [67,68], and NKN-CaTiO_3_ [69]) have received considerable attention mainly for two reasons: (i) piezoelectric properties exist over a wide range of temperature, and (ii) there are several possibilities for substitution and additions. An interesting possibility arises from the fact that if one can find a high piezoelectric performance lead–free ceramics and combine it with magnetostrictive material with high piezo-magnetic coefficient to achieve environment friendly magnetoelectric materials with desired sensitivity.

We have conducted extensive studies on phase transitions, synthesis, and piezoelectric/dielectric properties of (1 − x)(Na_0.5_K_0.5_)NbO_3_-xBaTiO_3 _ceramics (0.0 ≤ x ≤ 1.0). This system exhibits three phase transition regions corresponding to orthorhombic, tetragonal, and cubic phases. The composition 0.95(Na_0.5_K_0.5_)NbO_3_-0.05BaTiO_3_, which lies on boundary of orthorhombic and tetragonal phase, was found to exhibit excellent piezoelectric properties. The properties of this composition were further improved by addition of various additives making it suitable for multi-layer actuator application. The composition 0.06(Na_0.5_K_0.5_)NbO_3_-0.94BaTiO_3_ was found to lie on the boundary of tetragonal and cubic phase. This composition exhibited the microstructure with small grain size and excellent dielectric properties suitable for multi-layer ceramic capacitor application. In the next section, we summarize the progress made in the field of lead-free piezoelectric materials.

## 2. Progress on Lead–Free Piezoelectric Materials

Most of the investigated lead-free piezoelectric systems are based on Na_0.5_Bi_0.5_TiO_3_ (NBT) and K_0.5_Na_0.5_NbO_3_ (NKN) compositions. This section briefly describes the progress made on these two lead-free piezoelectric materials.

### 2.1. NBT-BT Lead-Free Piezoelectric

Na_0.5_Bi_0.5_TiO_3_ (NBT) was discovered by Smolenskii [70] in 1961. It was reported that NBT is a relaxor ferroelectric with diffuse phase transition from rhombohedral to tetragonal phase between 200 °C and 320 °C and from tetragonal to cubic phase at 540 °C [71,72,73,74]. The region between 200–320 °C has been subject of intense discussions related to the existence of antiferroelectric phase. Some researchers have suggested coexistence of rhombohedral and tetragonal phases in this temperature range with polar nano regions [75]. However in a recent work, *in situ* temperature dependent TEM studies showed phase transition from ferroelectric rhombohedral to anti-ferroelectric orthorhombic phase proceeded via an antiferroelectric modulated phase consisting of orthorhombic sheets in a rhombohedral matrix in the temperature range from 200–300 °C. A second phase transition from O-T phase occurs near 320 °C, which corresponds to the antiferroelectric/paraelectric phase transition. 

**Figure 2:**
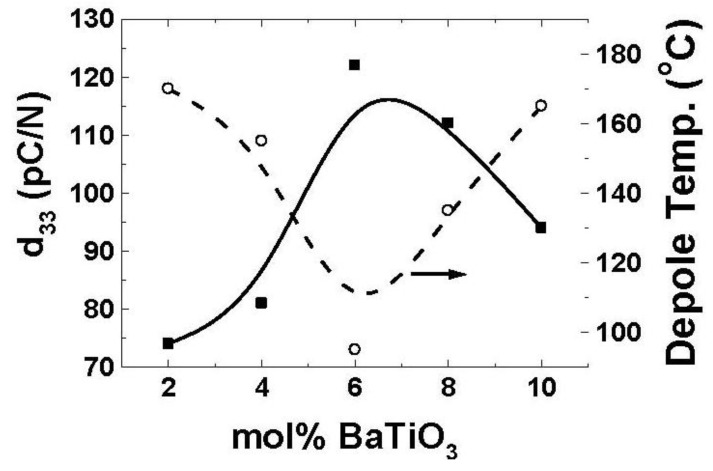
Variation of the piezoelectric constant and transition temperature (ferroelectric to antiferroelectric phase mentioned as depolarization temperature) as a function of BaTiO_3_ concentration in NBT–BT system.

The solid solution of NBT with various tetragonal compounds has been investigated including K_1/2_Bi_1/2_TiO_3_ (KBT), BaTiO_3_, CaTiO_3_, SrTiO_3_ and PbTiO_3_ [76,77,78,79]. Takenaka *et al*. [79] presented the phase diagram of NBT-BT showing the existence of a morphotropic phase boundary (MPB). The longitudinal piezoelectric constant (d_33_) for NBT-6% BT composition was found to be 125 pC/N along with k_33_ of 55% and loss tangent factor of 1.3%. However, Curie temperature (T_c_) and ferroelectric to anti-ferroelectric transition temperature (*T*_d_, also referred as depoling temperature) decreased at MPB. In comparison to lead-based perovskites, NBT has higher elastic modulus (~110 GPa *vs*. 70 GPa) and lower density (6 g/cm^3^) which makes it favorable for light-weight actuation applications [76]. Modification with BiScO_3_ and BiFeO_3_ lead to increment in T_c_ of NBT and NBT-BT up to 400 °C together with an improvement of remnant polarization (*P*_r_) [80,81,82]. Li *et al*. [83] have reported the dielectric and piezoelectric response of (1 − x)(Na_0.5_Bi_0.5_)TiO_3_-xNaNbO_3_ ceramics. The samples in composition range of 0.01 to 0.02 were found to exhibit d_33_ ~80–88 pC/N. In recent work, effect of Na non-stochiometry in Bi_0.5_Na_0.5-x_TiO_3_ ceramics was investigated [84] The grain size was found to decrease with increase in Na non-stoichiometry. However, d_33_ was increased from 74 pC/N (T_d_ ~190 °C) at x = 0.0 to 91 pC/N (T_d_ ~112 °C) at x = 3.5 and then dropped with further Na deficiency. The effect of bismuth excess on NBT-BT ceramics near MPB and compositions with x ≤ 0.505 was positive leading to large *P*_r_ ~37.5 − 41.1 µC/cm^2^ and d_33_ ~171–176 pC/N at RT with moderate depoling temperature (*T_d_*) ~85 °C [85]. Wang *et al*. [86] have studied (0.95 − x)(Bi_1/2_Na_1/2_)TiO_3_-x(Bi_1/2_K_1/2_)TiO_3_-0.05BaTiO_3_; x = 0–20 mol% compositions and specimen with x = 5 mol% were found to exhibit d_33_ ~148 pC/N, *k*_p_~34 %, *k*_t_~49.2 % and T_d_ ~125 °C. However, these modifiers also increased the magnitude of coercive field (E_c_) which makes the poling difficult. Generally, E_c_ and T_d_ are lower in doped materials, however cobalt doping was found to enhance T_d_ by 20 °C [87,88]. High piezoelectric properties in these Bi based compounds is always accompanied with the lowering of T_d_ as shown in Figure 2. Lower T_d_ leads to unstable domains which are easy to switch and hence give rise to higher piezoelectric constant [87]. Li doping of 4 at% in NBT-BT has been found to improve the piezoelectric coefficient as d_33_ = 176 pC/N, k_33_ = 0.6 and T_d_ = 171 °C [89]. In order to further improve the piezoelectric response without lowering the depoling temperature (T_d_) various researchers have used reactive template (RTGG) and template (TGG) grain growth method [90,91,92,93,94,95] to texture the ceramics. The <100>_c_ textured 0.94NBT-0.06BaTiO_3_ ceramics was found to exhibit *d*_33_ ~241 pC/N, *k*_p_ ~41.2 % and *k_t_* ~66.5% at RT with *T*_d_ ~115 °C. 

Investigations on growth of lead-free single crystals in NBT-BT systems has shown that near MPB compositions are congruently melting and can be grown by flux growth [96] and top seeded solution growth (TSSG) method [97]. Recently, longitudinal piezoelectric constant (d_33_) as high as 457 pC/N with k_33_ ~68.5 % was reported on Mn doped NBT-BT single crystal grown by TSSG method. Piezoelectric properties of various NBT-based materials are listed in Table 1 along with other prominent lead-free piezoelectric materials. In order to demonstrate practical feasibility, Chen *et al*. [98] investigated high frequency ultrasonic transducers with NBT-BT lead-free single crystal as the active element. The (001) oriented NBTBT crystal was found to exhibit a thickness mode electromechanical coupling coefficient k_t_ of ~0.52 and low clamped dielectric constant of ~80. 

Recently, giant electric field induced strain (~0.45%) was observed in (1 − x − y) (Bi_0.5_Na_0.5_)TiO_3_-xBaTiO_3_-y(K_0.5_Na_0.5_)NbO_3_ceramics [99,100,101]. The MPB composition 0.94(Bi_0.5_Na_0.5_)TiO_3_-0.06(BaTiO_3_) exhibited field induced ferroelectricity and remained ferroelectric after field removal [102]. On addition of KNN, these ceramics were found to exhibit giant strain due to the full recovery of original dimensions for every electric cycle [103]. It turned out that addition of KNN lowered the transition temperature (T_d_) [104]. Moreover, large electric field-induced strain in BNT-BT-KNN was attributed to a field induced transition from the antiferroelectric to the ferroelectric phase and explained in terms of change in the unit cell volume.

### 2.2. NKN Based Lead-Free Piezoelectric

**Figure 3:**
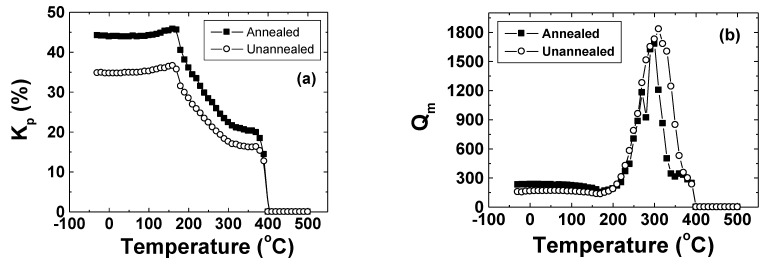
Temperature dependence of piezoelectric properties for KNN. **(a)** Radial mode coupling factor and **(b)** Mechanical quality factor.

Potassium sodium niobate (NaxK(1−x)NbO3), is considered a promising lead-free piezoelectric material with high Curie temperature exhibiting ferroelectric properties over a wide temperature range. It is well known that the composition corresponding to 0.5/0.5 in the NaNbO3–KNbO3 system, abbreviated as NKN, has the maximum in the piezoelectric properties. NKN undergoes a structural phase transformation sequence on cooling of paraelectric Cubic (C) →~415Co ferroelectric Tetragonal (T) →~210Co ferroelectric Orthorhombic (O) →~−150Co ferroelectric Rhombohedral (R). The T→O boundary is known as the polymorphic phase boundary (PPB) to designate its difference with the MPB. The PPB of KNN solutions is nearly independent of x, remaining unchanged in temperature for 0 < x < 1. This is in distinct contrast to the MPB for PZT, which is nearly independent of temperature, and fixed near x = 0.5. Figure 3 (a) and (b) shows the radial mode electromechanical coupling factor (k_p_) and mechanical quality factor (Q_m_) as a function of temperature for NKN. It can be clearly seen from this figure that piezoelectric properties remain almost constant until the FE_t_ phase appears at 180 °C. The magnitude of k_p_ at room temperature is of the order of 0.456 and Q_m_ is around 234. Since in this system the high temperature phase (FE_t_) is also ferroelectric there is no danger of depoling on exceeding the transition temperature. This provides a considerable advantage over the competing NBT-KBT and NBT-BT systems and for this reason KNN ceramics are the most promising high piezoelectric nonlead system.

NKN ceramics are difficult to sinter and exhibit poor aging characteristics in air. The volatile nature of constituent materials gives rise to phase instability at high temperature, as above 1100 °C alkali deficiency may result in formation of secondary phase exhibiting abnormal grain growth [105]. Hot pressed specimens have been found to show dense microstructure with theoretical density higher than 99% leading to higher coupling constant (~0.48) and d_33_ value (~160 pC/N) as compared to ceramics processed through conventional sintering [106,107,108]. Spark plasma sintering [109,110] has also been shown to provide high relative density of 98%, however d_33_ value was nominal with a magnitude in the vicinity of 158 pC/N. 

Piezoelectric properties of NKN based ceramics are a function of orthorhombic (O) to tetragonal (T) phase transition [111]. Ahn *et al.* [111] performed Rietveld and powder diffraction analysis to establish correlation between piezoelectric response, fraction of O and T phases, and NKN ratio for three different systems of (K,Na)NbO_3_-BaTiO_3_ (NKN-BT) NKN-LiNbO_3 _(LN), and (K,Na,Li)NbO_3_ (KNLN)-BT. It was found that higher piezoelectric properties in NKN based ceramics correlates with higher fraction of T phase and NKN ratio, where maximum piezoelectric properties are found in T-rich phase region with fraction of 70% and NKN ratio of 0.95. Several researchers [112,113] have studied the effect of the Na/K ratio in NKN system in order to improve the piezoelectric properties. Guo *et al*. [62] have studied the effect of Li-doping on piezoelectric properties of NKN by synthesizing the samples Li_x_(Na_0.5_K_0.5_)(1 − x)NbO_3_(NKLN), for x varying between 0.0 to 0.2. A sharp peak in piezoelectric properties (d_33_ ~235 pC/N, k_p_ ~44 %) was observed in the composition range of 0.05 < x < 0.07. Temperature dependent dielectric response of optimum composition revealed shifting of *T_c_* and *T_o-t_* in opposite directions. Zhao *et al*. [114] reported that Li-modified NKN ceramics exhibit orthorhombic to tetragonal phase transition, which is similar to morphotropic phase boundary (MPB) and hence exhibit high piezoelectric constant (d_33_~314 pC/N). Zhang *et al*. [115] substituted 0.058 mole % of Li^+^ on A-site while B-site was substituted with Sb^5+^ in range of 2–8 mol % resulting in drastic shifting of T_o–t_ to 60 °C. However, piezo-response was improved to d_33_ ~298 pC/N with k_p_ ~34.5%. Zhang *et al*. [64] in an extension of this work, synthesized the composition (Na_0.5_K_0.5_Nb)_(1−x) _(LiSb)_x_O_3_ with x = 0.048–0.056. The composition with x = 0.052 was found to exhibit optimum piezoelectric properties (d_33_ ~286, and k_p_ ~0.51). This improvement in electromechanical properties was attributed to lowering of T_o–t_ transition temperature. Some of the significant NKN based piezoelectric materials has been summarized in Table 1. 

**Table 1:** Dielectric and piezoelectric properties of the prominent lead-free systems.

Materials	d_33_	k_p_	k_33_	T_c_	T_o–t_/T_d_	Reference
BaTiO_3_	190	0.36	0.5	115	0	[116]
BT-BCN	330	0.43		80	--	[117]
NBT-KBT-LBT	216	0.401		350	160	[118]
NBT-KBT-BT	183	0.367	0.619	290	100	[119]
NBT-xBT; x = 6–8%	122–176	0.21–0.36		225–228	90–105	[87,120,121,122,123,124]
NBT-6BT+7.5L	208	0.368		260	85	[125,126]
NBT-6BT-2NKN	30			260		[100,101]
NBT-20KBT (MPB)	140–190	0.27–0.35		280–300	130–170	[127,128,129,130,131,132]
(K_0.5_Na_0.5_)NbO_3_ (H.P.)	127	0.46	0.6	420		[107,108]
(K_0.5_Na_0.5_)NbO_3_	80	0.35	0.51	420	195	[133]
NKN-Li (7%)	240	0.45	0.64	460	~20	[134]
NKN-LF4(Textured)	410	0.61	-	253	25	[91,135]
NKN-SrTiO3 (5%)	200	0.37	-	277	27	[67,68]
NKN-LiTaO3 (5%)	200	0.36	-	430	55	[63]
NKN-LiNbO3 (6%)	235	0.42	-	460	70	[62]
NKN-LiSbO_3_ (5%)	283	0.50	-	392	45	[64]

**Figure 4:**
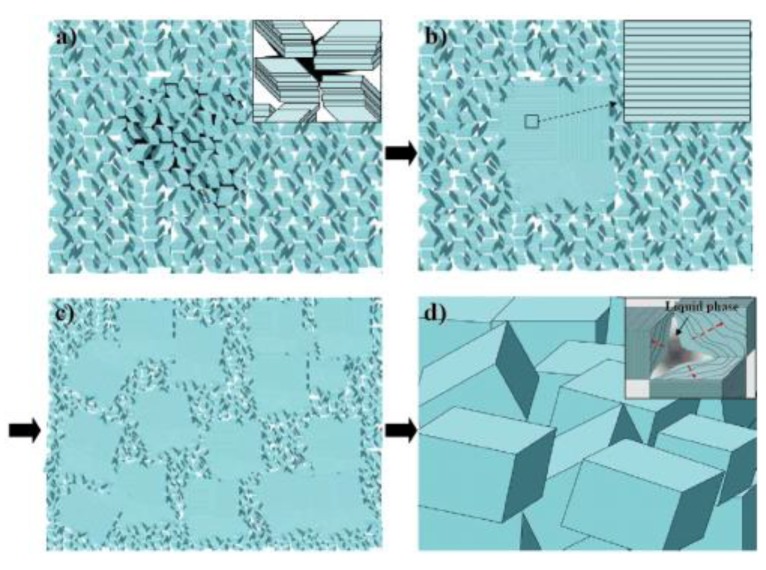
Schematic diagrams of sintering model as a function of temperature in KNLN-BT based ceramics.

**Figure 5:**
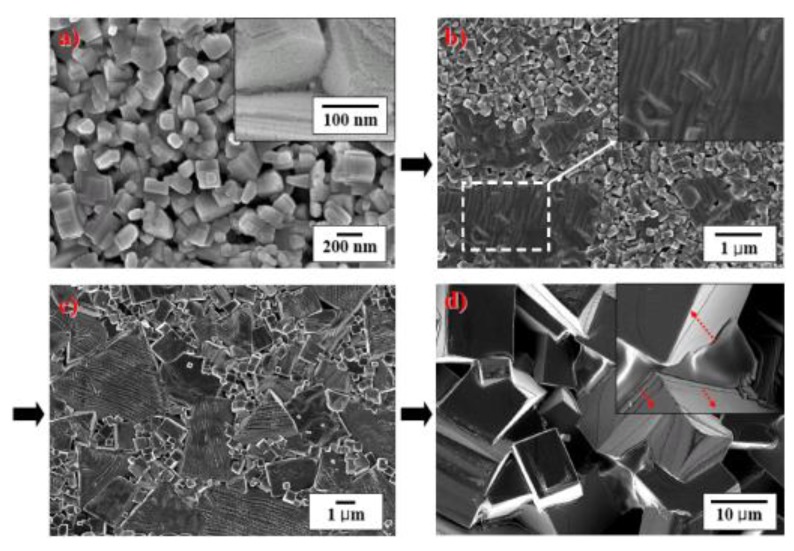
SEM images of the specimens sintered at various temperatures in KNLN-BT ceramics: **(a)** 950 °C; **(b)** 980 °C; **(c)** 1030 °C; and **(d)** 1080 °C.

In past years, we have extensively analyzed the sintering and grain growth processes in PPB systems. The microstructures of the compositions close to PPB were investigated in order to clarify the sintering behavior of the KNN based lead-free ceramics. Figure 4 illustrates the schematic diagram of our sintering model. The changes in the microstructure are shown as a function of temperature. Figure 5 shows the SEM images of the specimens sintered at various temperatures for the composition 0.995(K_0.48_Na_0.48_Li_0.04_)NbO_3_-0.005BaTiO_3_ (KLNN-BT). It has been shown before that KNN based ceramics exhibit cuboidal grains in the sintered microstructure. However, we found that the PPB composition ceramics rather consist of stacked structure of plate-like grains as schematically depicted in Figure 4 and experimentally observed in Figure 5. The rapid grain growth seen in this system was quantified by tracing the formation of liquid phase. These liquid phases are related to the formation of K_3_Li_2_Nb_5_O_15_ phase and Na-deficient KNLN-BT based phase formed by Na_2_O evaporation. The liquid phase redistributes between the plates under the influence of capillary stress gradients. The grain growth could be modeled by Ostwald ripening.

Recently in ABO_3_ type perovskite, an interesting trend between atomic weight ratio of A and B sites (R_w_ = W_A_/W_B_) and piezoelectric constant (d_33_) was demonstrated irrespective of tolerance factor (F_t_) [136]. Most of the piezoelectric compositions were shown to exhibit large response when *R_W_* for *A*-site heavy perovskites and 1/*R_W_* for *B*-site heavy perovskites was higher than 2.0. Using this rule, KNN-based [0.99(K_0.48_Na_0.48_Li_0.04_)(Nb_1−x_Sb_x_)O_3_ − 0.01BaTiO_3_] composition with d_33_ of 294 pC/N was designed. 

Various researchers have grown KNN based single crystals. Pure KNN single crystals exhibited d_33_ ~160 pC/N [137] and Mn doped KNN single crystals were reported to possess d_33_ ~270 pC/N, T_o–t_ ~193 °C and *T*c ~416 °C [137,138]. Recently, K_0.5_Na_0.5_NbO_3_-LiNbO_3_ (KKN-LN) single crystals were grown using Bridgeman technique by Xu *et al*. [139]. These KNN-LN single crystals exhibit d_33_ ~405 pC/N and k_t_ ~61% with T_c_ ~428 °C. However, composition fluctuation in single crystal is a critical issue and also their growth process is not cost-effective. Therefore, texturing of ceramics is gaining prominence as a potential alternative to single crystal. Saito *et al*. [91] reported that Li^+^, Ta^5+^ and Sb^5+^ substituted <001> oriented KNN exhibits d_33_ ~416 pC/N, but these substitutions shifts PPT from 200 °C to near room temperature (RT) giving rise to temperature dependent behavior. Recently, Chang *et al*. [140] reported that textured (K_0.5_Na_0.5_)(Nb_0.97_Sb_0.3_)O_3_ piezoelectric ceramics exhibits d_33_ ~208–218 pC/N, k_p_ ~0.64%, *T*_o–t_ ~160 °C and *T*_c_ ~352 °C, which was superior than random KNN based ceramics of same composition.

## 3. Modeling of Piezoelectric Materials

### 3.1. Single Domain Approximation in Computing the Piezoelectric Coefficients of Ferroelectrics

To explain the physical properties and ferroelectric transitions, the Landau-Ginsburg-Devonshire (LGD) phenomenological approach was successfully used. The free energy expansion coefficients are usually determined from experimental data on permittivity and spontaneous polarization. For this purpose, the first principle calculations are used. However, a more complete expansion of free energy density is necessary to estimate the physical parameters of some single crystals in a wide temperature range.

The main objective of this work is to investigate the influence of ferroelectric composition on dielectric and piezoelectric parameters. We consider PbTiO_3_, BaTiO_3_, LiTaO_3_, KNbO_3_, (Na_0.5_ K_0.5_)NbO_3_, and Na_0__.5_Bi_0__.5_TiO_3_-BaTiO_3_. We use the most appropriate expansions of free energy density to calculate the needed parameters.

#### 3.1.1. Lead Titanate 

Free energy density of a ferroelectric is defined by the expression:

*F = F_f_ + F_e_ + F_es_ + F_E_,* (5)

where *F_f_* is the ferroelectric ordering energy, *F_e_* is elastic energy, *F_es_* is electrostriction energy, and *F_E_* is the ferroelectric’s energy in an external electric field. The equilibrium polarization is assumed to be directed along the Z-axis. Using sixth order expansion for polarization components, the ferroelectric ordering energy takes the form:

(6) $$F_{f}=a_{1}P_{3}^{2}+a_{11}P_{3}^{4}+a_{111}P_{3}^{6}$$

where P is polarization, a_1_, . , a_111_ are the dielectric stiffness coefficients. Elastic energy is defined as:

(7) $$F_{e}=\frac{c_{11}}{2}(S_{1}^{2}+S_{2}^{2}+S_{3}^{2})+c_{12}(S_{1}S_{2}+S_{1}S_{3}+S_{2}S_{3})$$

where S_i _is the strain tensor component, c_11_ and c_12_ are the stiffness coefficients. Electrostriction energy can be written as follows:

(8) $$F_{es}=-q_{11}S_{3}P_{3}^{2}-q_{12}(S_{1}+S_{2})P_{3}^{2},$$

where q_11_ и q_12_ are the electrostriction coefficient. Ferroelectric’s energy in an applied electric field has the following form:

*F_E_ = −P_3_ E_3_* (9)

where E is the external electric field. Strain tensor components can be calculated by using the boundary conditions for mechanically free sample as:

(10) $$\left\{ \begin{array}{l} \frac{\partial F}{\partial S_{1}}=0,\\ \frac{\partial F}{\partial S_{3}}=0,\\ \frac{\partial F}{\partial S_{3}}=0 \end{array}\right.$$

Solving the set of equations (10) for strain components yields:

(11) $$\left\{ \begin{array}{l} S_{1}=\frac{P_{3}^{2}(-c_{12}q_{11}+c_{11}q_{12})}{-2{c_{12}}^{2}+c_{11}c_{12}+{c_{11}}^{2}},\\ S_{2}=\frac{P_{3}^{2}(-c_{12}q_{11}+c_{11}q_{12})}{-2{c_{12}}^{2}+c_{11}c_{12}+{c_{11}}^{2}},\\ S_{3}=\frac{(q_{11}c_{11}-2c_{12}q_{12}+c_{12}q_{11})P_{3}^{2}}{-2{c_{12}}^{2}+c_{11}c_{12}+{c_{11}}^{2}}. \end{array}\right.$$

Substituting the found expressions (11) into Equation (5) results in the following equation:

(12) $$F=a_{1}P_{3}^{2}+b\;P_{3}^{4}+a_{111}P_{3}^{6}-E_{3}P_{3},$$

where

$$b=\frac{2a_{11}{c_{11}}^{2}+2c_{11}a_{11}c_{12}-c_{11}{q_{11}}^{2}-2c_{11}{q_{12}}^{2}+4q_{12}c_{12}q_{11}-{q_{11}}^{2}c_{12}-4a_{11}{c_{12}}^{2}}{2(c_{11}+2c_{12})(c_{11}-c_{12})}.$$

It follows from Equation (12) that taking into account the boundary conditions for mechanically free sample results in re-normalization of ferroelectric stiffness coefficient for fourth order of polarization in the ferroelectric energy density. To find the equilibrium polarization of the sample, one should use the following equation:

(13) $$\frac{\partial F}{\partial P_{3}}=-E_{3}.$$

where F is defined by Equation (12). The solution of Equation (13) has the form:

(14) $$P_{3}=\frac{\sqrt{-\frac{3b-3\sqrt{b^{2}-3a_{111}a_{1}}}{a_{111}}}}{3}$$

Thus, the solution of Equations (10) and (13) enables finding of the equilibrium polarization and strain components that appear in Equation (5). Piezoelectric coefficients can be determined by using the equations for strains:

(15) $$d_{ik}=\frac{\partial S_{i}}{\partial P_{k}}\chi_{kk},$$

where $\chi_{kk}={\left(\frac{\partial^{2}F}{\partial P_{k}^{2}}\right)}^{-1}$ is dielectric susceptibility and, S_i_ are determined by Equation (11). Using Equations (15) and (11) we can find:

(16) $$d_{33}=\frac{2P_{3}(2q_{12}c_{12}-c_{11}q_{11}-c_{12}q_{11})}{\left(2{c_{12}}^{2}-{c_{11}}^{2}-c_{11}c_{12})(2a_{1}+12bP_{3}^{2}+30a_{111}P_{3}^{4}\right)},$$

(17) $$d_{31}=\frac{2P_{3}(c_{11}q_{12}-c_{12}q_{11})}{\left(2{c_{12}}^{2}-{c_{11}}^{2}-c_{11}c_{12})(2a_{1}+12bP_{3}^{2}+30a_{111}P_{3}^{4}\right)},$$

The permittivity can be found from the following equation:

(18) $$\varepsilon_{33}={\left(\frac{\partial^{2}F}{\partial {P_{3}}^{2}}\right)}^{-1}+\varepsilon_{0}$$

As an example, the parameters of lead titanate are estimated at room temperature Т = 25 °С. For calculations, the following coefficients were used in the free energy density function (in SI units) [141]: a_1_ = 3.8 × 10^5^ (Т − 479), a_11_ = −7.3 × 10^7^, a_111_ = 2.6 × 10^8^, *c*_11 _= 1.746 × 10^11^, *c*_12_ = 7.94 × 10^10^,* q*_11_ = 1.203 × 10^10^, *q*_12_ = −1.878 × 10^9^. According to our estimates, d_31_ = −11 × 10^12 ^m/V, d_33_ = 22 × 10^12^ m/V, ε_33_/ε_0_ = 75, Р_3_ = 1.5 С/m^2^. Experimentally measured piezoelectric coefficient is equal d_33_ = 25 × 10^12^ m/V [142] that is in agreement with calculated data.

#### 3.1.2. Barium Titanate

In an attempt to calculate the physical parameters of barium titanate using the ferroelectric energy density in the form of Equation (6), we obtained poor estimates that did not match the experimental data. Therefore, the expression for free energy density was expanded to eighth order for modeling the dielectric and piezoelectric parameters of barium titanate as:

(19) $$F_{f}=a_{1}P_{3}^{2}+a_{11}P_{3}^{4}+a_{111}P_{3}^{6}+a_{1111}P_{3}^{8}$$

where a_1111_ is the dielectric stiffness coefficient of eighth order. Performing the calculations similar to above enables reducing Equation (5) to the form:

(20) $$F=a_{1}P_{3}^{2}+b\;P_{3}^{4}+a_{111}P_{3}^{6}+a_{1111}P_{3}^{8}-E_{3}P_{3},$$

where *b* is defined by Equation (12). Based on Equation (13), the equilibrium polarization can be reduced to following form:

(21) P3=123a1111(r1−3(8 ba1111−3a1112)r1−3a111)12

where

r1={108a1111( ba111−2 a1a1111)−27 a1113+123[32 b3a1111−−9 b2a1112−108a1a1111(ba111−a1a1111)+27a1a1113]12a1111}13.

Using Equation (15), the piezoelectric coefficients can be derived as:

(22) $$d_{33}=\frac{2P_{3}(2q_{11}c_{11}-c_{12}q_{11}-2c_{12}q_{12})}{\left(c_{11}c_{12}-2{c_{12}}^{2}+{c_{11}}^{2})(2a_{1}+12bP_{3}^{2}+30a_{111}P_{3}^{4}+56a_{1111}P_{3}^{6}\right)},$$

(23) $$d_{31}=\frac{2P(-c_{12}q_{11}+c_{11}q_{12})}{\left(c_{11}c_{12}-2{c_{12}}^{2}+{c_{11}}^{2})(2a_{1}+12bP_{3}^{2}+30a_{111}P_{3}^{4}+56a_{1111}P_{3}^{6}\right)},$$

Numerical estimates for dielectric and piezoelectric parameters were carried out at room temperature Т = 25 °С. These estimates depend on following coefficients that enter the expression for free energy density [143]: *a*_1_ = 4.124 × 10^5^ (T − 115), *a*_11_ = 5.328 × 10^8^, *a*_111_ = 1.294 × 10^9^, *a*_1111_ = 3.863 × 10^10^, *c*_11_ = 1.755 × 10^11^, *c*_12_ = 8.464 × 10^10^, *q*_11_ = 1.203 × 10^10^, *q*_12_ = −1.878 × 10^9^. The calculated values are listed below: d_31_ = −37 × 10^−12^ m/V, d_33_ = 95 × 10^−12^ m/V, ε_33_/ε_0_ = 189, Р_3_ = 0.26 С/m^2^. The obtained estimates are in full agreement with measurement data which were reported recently [144]: d_31_ = −29.4 × 10^−12^ m/V, d_33_ = 86.3 × 10^−12^ m/V, ε_33_/ε_0_ = 188, Р_3_ = 0.26 С/m^2^.

#### 3.1.3. LiTaO_3_

LiNbO_3_ and LiTaO_3_ belong to the 3*m* point group. Denoting the crystallographic uniaxial directions as the *z*-axis, the free-energy expansion is given as:

(24) $$\begin{array}{l} F=\frac{a_{1}P_{3}^{2}}{2}+\frac{a_{2}P_{3}^{4}}{4}+b_{1}{S_{3}}^{2}+b_{2}{\left(S_{1}+S_{2}\right)}^{2}+\\ +b_{3}({\left(S_{1}-S_{2}\right)}^{2}{S_{6}}^{2})+b_{4}S_{3}(S_{1}+S_{2})+b_{5}({S_{4}}^{2}+{S_{5}}^{2})+\\ +b_{6}((S_{1}-S_{2})S_{4}+S_{5}S_{6})+g_{1}(S_{1}+S_{2})P_{3}^{2}+g_{2}S_{3}P_{3}^{2} \end{array}$$

where *b_i_* and *g_i_* are stiffness and electrostriction coefficients. Equilibrium strain components take the form for free sample as:

(25) $$\begin{array}{l} S_{4}=0,\\ S_{6}=0,\\ S_{5}=0,\\ S_{2}=\frac{(-2b_{1}g_{1}+g_{2}b_{4})P_{3}^{2}}{2({b_{4}}^{2}-4b_{1}b_{2})},\\ S_{3}=\frac{(b_{4}g_{1}+2g_{2}b_{2})P_{3}^{2}}{{b_{4}}^{2}-4b_{1}b_{2}},\\ S_{1}=\frac{(-2b_{1}g_{1}+g_{2}b_{4})P_{3}^{2}}{2({b_{4}}^{2}-b_{1}b_{2})}. \end{array}$$

Substituting Equation (25) into Equation (24) yields:

(26) $$F=a_{1}P_{3}^{2}+a_{0}\;P_{3}^{4},$$

where $a_{0}=\frac{a_{2}b_{4}^{2}-4g_{1}g_{2}b_{4}+4b_{1}g_{1}^{2}-4a_{2}b_{1}b_{2}+4b_{2}g_{2}^{2}}{b_{4}^{2}-4b_{1}b_{2}}.$

Equation (26) enables to find the equilibrium polarization:

(27) $$P_{3}=\sqrt{\frac{a_{1}}{a_{0}}}$$

Then the expressions for piezoelectric coefficients are as follows:

(28) $$\begin{array}{l} d_{33}=-\frac{2P_{3}(b_{4}g_{1}-2b_{2}g_{2})}{\left({b_{4}}^{2}-4b_{1}b_{2})(-a_{1}+3a_{0}P_{3}^{2}\right)},\\ d_{31}=-\frac{(2b_{1}g_{1}-b_{4}g_{2})P_{3}}{\left({b_{4}}^{2}-4b_{1}b_{2})(-a_{1}+3a_{0}P_{3}^{2}\right)}. \end{array}$$

For numerical estimation we used the following LGD potential coefficients for LiTaO_3_: *a_1_* = 0.1256 × 10^10^, *b_1_* = 0.1355 × 10^12^, *b_2_* = 0.6475 × 10^11^, *b_3_* = 0.4925 × 10^11^, *b_4_* = 0.74 × 10^11^,* b_5_* = 0.48 × 10^11^, *b_6_* = −0.12 × 10^11^, *g_1_* = −0.202 × 10^9^, *g_2_* = 0.1317 × 10^10^, a*_2_* = 0.5043 × 10^10^. The calculated values were found to be: d_31_ = 1.0 × 10^−12^ m/V, d_33_ = −2.5 × 10^−12^ m/V, ε_33_/ε_0_ = 46, Р_3_ = 0.5 С/m^2^.

#### 3.1.4. KNbO_3_

To perform the thermodynamic analysis of KNbO_3_, polarization was assumed to be P = (P_3_, 0, P_3_) for the rhombohedral phase which is stable at room temperature. In this case, the ferroelectric ordering energy takes the following form under stress-free condition [145,146,147,148]:

(29) $$F_{f}=2a_{1}P_{3}^{2}+(2a_{11}+a_{12})P_{3}^{4}+2(a_{111}+a_{112})P_{3}^{6}+(2a_{1111}+2a_{1112}+a_{1122})P_{3}^{8}$$

One can see that Equation (29) is similar to Equation (19) and differs from the latter in renormalized coefficients. Taking this into account, we can calculate the spontaneous polarization components and dielectric susceptibility as:

(30) $$\chi_{ki}={\left(\frac{\partial^{2}F}{\partial P_{k}\partial P_{i}}\right)}^{-1}$$

When the cell axis is along the pseudocubic direction, the spontaneous strains can be expressed as:

(31) $$\begin{array}{l} S_{11}=Q_{11}P_{1}^{2}+Q_{12}(P2+P_{3}^{2});\\ S_{22}=Q_{11}P_{2}^{2}+Q_{12}(P_{1}^{2}+P_{3}^{2});\\ S_{33}=Q_{11}P_{3}^{2}+Q_{12}(P_{2}^{2}+P_{1}^{2}); \end{array}$$

where Q_11 _and Q_12_ are the electrostrictive coefficients. Finally, the piezoelectric coefficients can be found from Equation (15). The energy expansion coefficients used for estimates (in SI units) were as follows: a_1_ = 4.273 × 10^5^(T − 377), a_11_ = 6.36 × 10^8^, a_12_ = 9.66 × 10^8^, a_111_ = 2.81 × 10^9^, a_112_ = −1.99 × 10^9^, a_123_ = 6.03 × 10^9^, a_1111_ = 1.74 × 10^10^, a_1112_ = 5.99 × 10^9^, a_1122_ = 2.50 × 10^10^, a_1123_ = −1.17 × 10^10^, Q_11_ = 0.12, Q_12_ = −0.053, Q_44_ = 0.052. The calculated values are at room temperature were: P_s_ = 0.45 С/m^2^, ε_33_/ε_0_ = 55, ε_22_/ε_0_ = 160, ε_11_/ε_0_ = 1000, d_31_ = 3.4 × 10^−12^ m/V, d_32_ = −24.3 × 10^−12 ^m/V, d_33_ = 27.4 × 10^−12^ m/V.

**Table 2:** Dielectric stiffness coefficients used for estimates (in SI units).

Coefficients	PbTiO_3_	BaTiO_3_	LiTaO_3_	KNbO_3_	(Na,K)NbO_3_	Na_0.5_Bi_0.5_TiO_3_-BaTiO_3_
a_1_	3.8 × 10^5^ (Т − 479)	4.124 × 10^5^ (T − 115)	1.256 × 10^9^	4.273 × 10^5^ (T − 377)		
a_11_ (a_2_)	−7.3 × 10^7^	5.328 × 10^8^	5.043 × 10^9^	6.36 × 10^8^		
a_12_				9.66 × 10^8^		
a_111_	2.6 × 10^8^	1.294 × 10^9^		2.81 × 10^9^		
a_112_				−1.99 × 10^9^		
a_123_				6.03 × 10^9^		
a_1111_		3.863 × 10^10^		1.74 × 10^10^		
a_1112_				5.99 × 10^9^		
a_1122_				2.50 × 10^10^		
a_1123_				−1.17 × 10^10^		

**Table 3:** Calculated dielectric and piezoelectric parameters (in SI units).

B	PbTiO_3_		BaTiO_3_		LiTaO_3_		KNbO_3_		(Na,K)NbO_3_		Na_0.5_Bi_0.5_TiO_3_-BaTiO_3_	
	Theory	Obser-vation	Theory	Obser-vation	Theory	Obser-vation	Theory	Obser-vation	Theory	Obser-vation	Theory	Obser-vation
P_s_ (С/m^2^)	1.5		0.26	0.26	0.5		0.45	0.42				
ε_33_/ε_0_	75		189	188	46		55					
ε_22_/ε_0_							160					
ε_11_/ε_0_							1000					
d_31 _(10^−12^m/V)	−11		−37	−29.4	1.0		3.4	9.8				
d_32 _(10^−12^m/V)							−24.3	−22.3				
d_33 _(10^−12^m/V)	22	25	95	86.3	−2.5		27.4	29.3				

Dielectric stiffness coefficients and calculated dielectric parameters for some piezoelectric materials have been summarized in Table 2 and Table 3. One can conclude that substitution of Pb by Ba in PbTiO_3_ enables higher values of permittivity and piezoelectric coupling coefficients. LiTaO_3 _and KNbO_3 _are characterized by weaker piezoelectric coupling. In summary, electrical parameters of lead-based and lead-free piezoelectrics such as the polarization, dielectric permittivity and piezoelectric coefficients can be determined using the Equations (14), (15) and (18) and experimental dielectric stiffness coefficients.

### 3.2. Theory of Ferroelectric Solid Solutions (Generalized Lattice Model) [149,150,151,152]

One of the most common methods for modifying the physical properties is by introduction of some impurities into ferroelectric compounds. Thus, the theoretical and experimental investigations of ferroelectric solid solutions are very attractive. There are a few different approaches to address the problem of theoretically modeling the ferroelectric solid solutions. Most popular approaches are based on density functional method, quantum chemical calculation and phenomenological thermodynamic models (such as Landau-Ginsburg-Devonshire model). This section contains application of the lattice model to ferroelectric solid solution. This model permits all calculations in analytical form with satisfactory resemblance to experimental data.

Let us consider ferroelectric solid solution as a lattice with two kinds of dipoles distributed over the sites. Suppose the energy of two dipoles located in sites $R_{i}$ and $R_{j}$ has the following form:

(32) $$U_{ij}=-\sum_{\alpha ,\gamma }\;Q_{\alpha \gamma }\left(R_{i}-R_{j}\right)\;\left(D_{\alpha }\left(R_{i}\right)\cdot D_{\gamma }\left(R_{j}\right)\right)\;n_{\alpha }\left(R_{i}\right)\;n_{\gamma }\left(R_{j}\right),$$

where $n_{\alpha }\left(R_{i}\right)$ is a dichotomous random variable with values 1 and 0 : it is equal to 1 if site $R_{i}$ contains a particle of α -th component, and 0 otherwise.

These variables $n_{1}\left(R_{i}\right)$ and $n_{2}\left(R_{i}\right)$ obey the relation:

(33) $$n_{1}\left(R_{i}\right)+n_{2}\left(R_{i}\right)=1\text{,}$$

their mean values are:

(34) $$<n_{1}\left(R_{i}\right)>=c_{1},\;<n_{2}\left(R_{i}\right)>=c_{2},\;c_{1}+c_{2}=1$$

where $c_{1}$ , $c_{2}$ are component’s concentrations in the solution.The functions $Q_{\alpha \gamma }\left(R\right)$ in lattice models are long-range interaction potentials with cutting on short distances. A system of dipoles in presence of an external electric field E has the following Hamiltonian:

(35) $$\begin{array}{r} H=-\frac{1}{2}\sum_{i,j}\;\sum_{\alpha ,\gamma }\;Q_{\alpha \gamma }\left(R_{i}-R_{j}\right)\;\left(D_{\alpha }\left(R_{i}\right)\cdot D_{\gamma }\left(R_{j}\right)\right)\;n_{\alpha }\left(R_{i}\right)\;n_{\gamma }\left(R_{j}\right)\\ -\sum_{i,\alpha }\left(E\cdot D_{\alpha }\left(R_{i}\right)\right)\;n_{\alpha }\left(R_{i}\right). \end{array}$$

In self-consistent field approximation for the long-range parts interactions this Hamiltonian can be transformed to following form:

(36) $$H=-\sum_{\alpha }\;\left\{\sum_{i}\left(E_{\alpha }^{\text{eff}}\cdot D_{\alpha }\left(R_{i}\right)\right)\;n_{\alpha }\left(R_{i}\right)\right\}=\sum_{\alpha }\;{\tilde{H}}_{\alpha }^{\text{eff}}\;,$$

where

(37) $$E_{\alpha }^{\text{eff}}=E+\frac{1}{2}\;<\sum_{j,\gamma }\;Q_{\alpha \gamma }\left(R_{i}-R_{j}\right)\;D_{\gamma }\left(R_{j}\right)\;n_{\gamma }\left(R_{j}\right)>\text{.}$$

The second term $\frac{1}{2}\;<\sum_{j,\gamma }\;Q_{\alpha \gamma }\left(R_{i}-R_{j}\right)\;D_{\gamma }\left(R_{j}\right)\;n_{\gamma }\left(R_{j}\right)>$ in this formula is the local effective field due to all the dipoles at point $R_{i}$ for dipole of α -th kind (in general case the effective fields for the components are not identical).

#### 3.2.1. Effective Fields

To evaluate Equation (37) one should find the mean values $<n_{\gamma }\left(R_{j}\right)>$ under the sum over j,γ sign. In general case these mean values depend on the correlations between component distributions, but in the *effective field approximation* these mean values depend on concentrations of the components:

(38) $$<n_{\gamma }\left(R_{j}\right)>=c_{\gamma }.$$

As a result we have the following expression for effective field

(39) $$E_{\alpha }^{\text{eff}}=E+\frac{1}{2}\;\sum_{\gamma }\;Q_{\alpha \gamma }^{\left(0\right)}<D_{\gamma }>c_{\gamma },$$

where

(40) $$Q_{\alpha \gamma }^{\left(0\right)}=\sum_{j}\;Q_{\alpha \gamma }\left(R_{i}-R_{j}\right)=\sum_{j}\;Q_{\alpha \gamma }\left(R_{j}\right)$$

(the last relation holds by virtue of translation invariance of crystal).

#### 3.2.2. Generating Functional and Polarization of Solution

Generating functional (*i.e*., the partition function a system at an external field presence) in effective field approximation has the following form:

(41) $$Z=\frac{1}{N_{1}!\;N_{2}!}\int \cdots \int \left[\prod_{i,\alpha }\frac{d\Omega_{i,\alpha }}{4\pi }\right]\;exp\left[\beta \sum_{i,\alpha }\;\left(E_{\alpha }^{\text{eff}}\cdot D_{\alpha }\left(R_{i}\right)\right)n_{\alpha }\left(R_{i}\right)\right],$$

where $d\Omega_{i,\alpha }$ is a solid angle infinitesimal element in spherical coordinates $\left(D_{\alpha },\theta_{i},\phi_{i}\right)$

(42) $$d\Omega_{i,\alpha }=sin\theta_{i}\;d\theta_{i}\;d\phi_{i},$$

β=1/T , T is absolute temperature in energetic units, $N_{1}$ and $N_{2}$ are numbers of the dipoles.

The multiple integral (41) is a product of the same type integrals

(43) $$\int \;\frac{d\Omega }{4\pi }\;exp\left[\beta E^{\text{eff}}Dcos\theta \right]=\frac{\sinh\left(\beta E^{\text{eff}}D\right)}{\beta E^{\text{eff}}D},$$

therefore generating functional in mean field approximation can be evaluated easily

(44) $$Z\;=\;\frac{1}{N_{1}!\;N_{2}!}\;{\left[\frac{\sinh\left(\beta E_{1}^{\text{eff}}D_{1}\right)}{\beta E_{1}^{\text{eff}}D_{1}}\right]}^{N_{1}}\;{\left[\frac{\sinh\left(\beta E_{2}^{\text{eff}}D_{2}\right)}{\beta E_{2}^{\text{eff}}D_{2}}\right]}^{N_{2}}.$$

Hence we find the system polarization (i.e dipole moment per volume unit) as:

(45) $$\begin{array}{r} P\;=\;\frac{1}{V\beta }\;\frac{\partial lnZ}{\partial E}\;\\ =\left\{\frac{N_{1}}{V}\left[\coth\left(\beta E_{1}^{\text{eff}}D_{1}\right)-\frac{1}{\left(\beta E_{1}^{\text{eff}}D_{1}\right)}\right]D_{1}+\frac{N_{2}}{V}\left[\coth\left(\beta E_{2}^{\text{eff}}D_{2}\right)-\frac{1}{\left(\beta E_{2}^{\text{eff}}D_{2}\right)}\right]D_{2}\right\}. \end{array}$$

Thus, full polarization of binary solid solution contains two contributions related to both of the components, but these contributions are mutually dependent via the effective fields. Therefore the components contributions into polarization are not additive.

#### 3.2.3. Mean Value of a Single Particle Dipole Moment in Effective Field

Mean value of a single particle dipole moment in external field E defined as:

(46) $$<D>=\frac{\int \;e^{-\beta H_{1}}\;D\;cos\theta \;d\Omega_{1}}{\int \;e^{-\beta H_{1}}\;d\Omega_{1}},$$

and has well known result [153,154,155]:

(47) <D>=D L(βDE),

where

(48) $$L\left(z\right)=\left[\coth\left(z\right)-\frac{1}{z}\right]$$

is the Langevin function, $H_{1}$ is the Hamiltonian of single dipole with moment D in external field E

(49) $$H_{1}=-\left(E\cdot D\right).$$

For one-component the system of expressions (37) has a simple form

(50) $$E^{\text{eff}}=E+\frac{1}{2}\;Q^{\left(0\right)}<D>,$$

Substitution $E^{\text{eff}}$ instead of E into formula (43) leads to transcendent equation with respect to <D> . It is well known this equation has a nontrivial solution <D>≠0 at $T<T_{c}$ .

#### 3.2.4. Polarization of Solid Solution

System of equations for mean values $<D_{1}>$ and $<D_{2}>$ of dipole moments of the solution components follows from relation (45):

(51) $$\{\begin{array}{l} <D_{1}>=D_{1}\left[\coth\left(\beta D_{1}\left\{E+\frac{1}{2}\sum_{\gamma }Q_{1\gamma }^{\left(0\right)}\;<D_{\gamma }>\;c_{\gamma }\right\}\right)-\frac{1}{\beta D_{1}\left\{E+\frac{1}{2}\sum_{\gamma }Q_{1\gamma }^{\left(0\right)}\;<D_{\gamma }>\;c_{\gamma }\right\}}\right]\\ <D_{2}>=D_{2}\left[\coth\left(\beta D_{2}\left\{E+\frac{1}{2}\sum_{\gamma }Q_{2\gamma }^{\left(0\right)}\;<D_{\gamma }>\;c_{\gamma }\right\}\right)-\frac{1}{\beta D_{2}\left\{E+\frac{1}{2}\sum_{\gamma }Q_{2\gamma }^{\left(0\right)}\;<D_{\gamma }>\;c_{\gamma }\right\}}\right]. \end{array}$$

Note the connection between $<D_{1}>$ and $<D_{2}>$ realize via non-diagonal elements of matrix $Q_{\alpha \gamma }^{\left(0\right)}$ only. It is clear that the solution $\left\{<D_{1}>,<D_{2}>\right\}$ of this system of equations depends on the temperature, the external field, and all of the matrix elements $Q_{\alpha \gamma }^{\left(0\right)}$ . To solve this system we should know the parameters of the physical system, *i.e*., matrix elements $Q_{\alpha \gamma }^{\left(0\right)}$ . These elements should be find using some experimental data.

Solution of system (51) with respect to $<D_{1}>$ , $<D_{2}>$ permits to find polarization of the system as function of external field E

(52) $$P=\frac{N}{V}\;\left[c_{1}\;<D_{1}>+c_{2}\;<D_{2}>\right]$$

and consequently the susceptibility χ of this system:

(53) $$\chi =\frac{N}{V}\;\left[c_{1}\;\frac{\partial <D_{1}>}{\partial E}+c_{2}\;\frac{\partial <D_{2}>}{\partial E}\right]$$

The Langevin function L(z) contains two terms. The first term is a transcendent function, the second is an algebraic function. Both of them have a singularity at z=0 . These circumstances complicate search of the solution. Therefore, the Langevin function should be approximated by some more suitable function with correct asymptotic behavior at z→0 and z→∞ . In the capacity of such approximation we shall use the following function

(54) $$L(z)\approx \frac{2}{\pi }\arctan\left(\frac{\pi \;z}{6}\right).$$

Note, this approximation is well not only for the Langevin function, but also for its derivative

(55) $$L^{\;'}(z)\approx \frac{12}{36+\pi^{2}z^{2}}.$$

This approximation for the Langevin function permits to simplify the system of Equations (51)

(56) $$<D_{\alpha }>=\frac{2\;D_{\alpha }}{\pi }\arctan\left(\frac{\pi \beta D_{\alpha }}{6}\left[E+\frac{1}{2}\sum_{\gamma }Q_{\alpha \gamma }^{\left(0\right)}\;<D_{\gamma }>\;c_{\gamma }\right]\right),\;(\alpha =\text{1,2}).$$

#### 3.2.5. Parameters of the Components

Some of the parameters $D_{\alpha }$ and $Q_{\alpha \alpha }^{\left(0\right)}$ of solution can be find from experimental data of the components. Equation (56) for a pure component ( $c_{\alpha }=1,\;{c_{\gamma }|}_{\gamma \neq \alpha }=0$ ) have the following form

(57) $$<D_{\alpha }>=\frac{2\;D_{\alpha }}{\pi }\arctan\;\left(\frac{\pi \beta D_{\alpha }}{6}\left\{E+\frac{1}{2}Q_{\alpha \alpha }^{\left(0\right)}\;<D_{\alpha }>\right\}\right).$$

This equation describes connection between external field E and mean value of dipole moment $D_{\alpha }$ . Critical temperature can find from condition existence of nontrivial solution (*i.e*., $<D_{\alpha }>\neq 0$ ) in external field vanishing E=0 . Graphical analysis of Equation (57) leads to following connection between critical temperature $T_{c}^{\alpha }$ and the model parameters

(58) $$\frac{{\left(D_{\alpha }\right)}^{2}\;Q_{\alpha \alpha }^{\left(0\right)}}{6}=T_{c}^{\alpha }\;.$$

Differentiating both of sides of Equation (57) with respect to E

(59) $$\frac{\partial <D_{\alpha }>}{\partial E}=\frac{12\beta {\left(D_{\alpha }\right)}^{2}}{36+{\left(\pi \beta D_{\alpha }\right)}^{2}\;{\left[E+\frac{1}{2}Q_{\alpha \alpha }^{\left(0\right)}<D_{\alpha }>\right]}^{2}}\;\left(1+\frac{1}{2}Q_{\alpha \alpha }^{\left(0\right)}\frac{\partial <D_{\alpha }>}{\partial E}\right),$$

we find derivate

(60) $$\frac{\partial <D_{\alpha }>}{\partial E}=\frac{12\beta {\left(D_{\alpha }\right)}^{2}}{\left[36+{\left(\pi \beta D_{\alpha }\right)}^{2}\;{\left[E+\frac{1}{2}Q_{\alpha \alpha }^{\left(0\right)}<D_{\alpha }>\right]}^{2}\right]\;-6\beta Q_{\alpha \alpha }^{\left(0\right)}{\left(D_{\alpha }\right)}^{2}}.$$

Eliminating $Q_{\alpha \alpha }^{\left(0\right)}$ from the last equation with account (58), we obtain susceptibility of the pure ferroelectrics:

(61) $$\chi =\frac{N_{\alpha }}{V}\;\frac{\partial <D_{\alpha }>}{\partial E}=\frac{N_{\alpha }}{V}\;\frac{12\beta {\left(D_{\alpha }\right)}^{2}}{\left\{36\left(1-\frac{T_{c}}{T}\right)+{\left(\frac{\pi }{T}\right)}^{2}\;{\left[ED+3T_{c}\frac{<D_{\alpha }>}{D_{\alpha }}\right]}^{2}\right\}}.$$

At E=0 the susceptibility have a singularity in vicinity of the critical point $T_{c}$ .

#### 3.2.6. Susceptibility of Solid Solution

After differentiating both sides of each equation in (56) over E we obtain a system of linear algebraic equations for $\frac{\partial <D_{\alpha }>}{\partial E}$ :

(62) $$\frac{\partial <D_{\alpha }>}{\partial E}=\frac{12\beta {\left(D_{\alpha }\right)}^{2}}{36+{\left(\pi \beta D_{\alpha }\right)}^{2}\;{\left[E+\frac{1}{2}\sum_{\gamma }Q_{\alpha \gamma }^{\left(0\right)}c_{\gamma }<D_{\gamma }>\right]}^{2}}\;\left(1+\frac{1}{2}\sum_{\gamma }Q_{\alpha \gamma }^{\left(0\right)}c_{\gamma }\frac{\partial <D_{\gamma }>}{\partial E}\right).$$

This system have the following short form:

(63) $$\{\begin{array}{l} \left(1-A_{1}B_{11}\right)\;\frac{\partial <D_{1}>}{\partial E}-A_{1}B_{12}\frac{\partial <D_{2}>}{\partial E}=A_{1};\\ -A_{2}B_{21}\frac{\partial <D_{1}>}{\partial E}+\left(1-A_{2}B_{22}\right)\;\frac{\partial <D_{2}>}{\partial E}=A_{2}, \end{array}$$

where

(64) $$A_{\alpha }=\frac{12\beta {\left(D_{\alpha }\right)}^{2}}{36+{\left(\pi \beta D_{\alpha }\right)}^{2}\;{\left[E+\frac{1}{2}\sum_{\gamma }Q_{\alpha \gamma }^{\left(0\right)}c_{\gamma }<D_{\gamma }>\right]}^{2}},$$

and

(65) $$B_{\alpha \gamma }=\frac{1}{2}Q_{\alpha \gamma }^{\left(0\right)}c_{\gamma }.$$

Hence we have for $\frac{\partial <D_{1}>}{\partial E}$ and $\frac{\partial <D_{2}>}{\partial E}$ :

(66) $$\{\begin{array}{l} \frac{\partial <D_{1}>}{\partial E}=\frac{A_{1}\left(1-A_{2}B_{22}+A_{2}B_{12}\right)}{1-A_{1}B_{11}-A_{2}B_{22}+A_{1}A_{2}\left[B_{11}B_{22}-B_{12}B_{21}\right]},\\ \frac{\partial <D_{2}>}{\partial E}=\frac{A_{2}\left(1-A_{1}B_{11}+A_{1}B_{21}\right)}{1-A_{1}B_{11}-A_{2}B_{22}+A_{1}A_{2}\left[B_{11}B_{22}-B_{12}B_{21}\right]}. \end{array}$$

This system of equations will be used for the critical point finding.

#### 3.2.7. Critical Points of Solid Solutions and Model Parameters

The solution susceptibility (53) in the critical point has a singularity due to vanishing denominators in right hand sides of (66)

(67) $$1-A_{1}B_{11}-A_{2}B_{22}+A_{1}A_{2}\left[B_{11}B_{22}-B_{12}B_{21}\right]=0.$$

This equation takes place under the conditions

(68) $$\{\begin{array}{l} E=0;\\ <D_{1}>=<D_{1}>=0. \end{array}$$

The second of these conditions due to the polarization vanishing at $T>T_{c}$ .

Substituting expressions (64) and (65) with conditions (68) account into Equation (67), we obtain the quadratic equation with respect to critical temperature T

(69) $$T^{2}-\frac{{\left(D_{1}\right)}^{2}Q_{11}^{\left(0\right)}c_{1}+{\left(D_{2}\right)}^{2}Q_{22}^{\left(0\right)}c_{2}}{6}\;T+\frac{c_{1}c_{2}}{36}{\left(D_{1}\right)}^{2}{\left(D_{2}\right)}^{2}\left[Q_{11}^{\left(0\right)}Q_{22}^{\left(0\right)}\;-\;Q_{12}^{\left(0\right)}Q_{21}^{\left(0\right)}\right]=0.$$

Discriminant of this equation in relation to symmetry property of the matrix $Q_{\alpha \gamma }^{\left(0\right)}$ is non-negative

(70) $$\tilde{D}={\left[{\left(D_{1}\right)}^{2}Q_{11}^{\left(0\right)}c_{1}-{\left(D_{2}\right)}^{2}Q_{22}^{\left(0\right)}c_{2}\right]}^{2}+4c_{1}c_{2}{\left(D_{1}\right)}^{2}{\left(D_{2}\right)}^{2}Q_{12}^{\left(0\right)}Q_{11}^{\left(0\right)}\geq 0,$$

therefore this equation has two real solutions.

Two variants are possible depending on the quantity Q sign

(71) $$Q=Q_{11}^{\left(0\right)}Q_{22}^{\left(0\right)}\;-\;Q_{12}^{\left(0\right)}Q_{21}^{\left(0\right)}.$$

• Q≤0 . In this case one of the solutions is positive, and the second solution is negative. The positive solution has a physical sense as the Curie temperature, the negative solution has not any physical sense. 

• Q>0 . In this case both of the solutions are positive. A physical interpretation of this case can be clarified after temperature analysis of the Equations (56)’s solutions. Thus, at condition Q>0 two-component ferroelectric solid solution has two critical points. 

Note that the critical points of solutions depends on such combinations dipole moments $D_{\alpha }$ and interatomic potentials $Q_{\alpha \gamma }^{\left(0\right)}$

(72) $$G_{\alpha \gamma }=Q_{\alpha \gamma }^{\left(0\right)}D_{\alpha }D_{\gamma }.$$

Transform the equation (65) using these combinations

(73) $$T^{2}-\frac{G_{11}\left(1-x\right)+G_{22}x}{6}\;T+\frac{x(1-x)}{36}\left[G_{11}G_{22}\;-\;G_{12}G_{21}\right]=0,$$

where $x=c_{2}$ is second component concentration, $c_{1}=1-x$ .

Let us introduce a new parameter

(74) $$G=G_{11}G_{22}\;-\;G_{12}G_{21}$$

and consider a general way of the model parameters $G_{11}$ , $G_{22}$ , G finding by the method of least squares.

Let us introduce the function $f\left(G,G_{11},G_{22},T,x\right)$

(75) $$f\left(G,G_{11},G_{22},T,x\right)=T^{2}-\frac{G_{11}\left(1-x\right)+G_{22}x}{6}\;T+\frac{x(1-x)}{36}G$$

and find the parameters $G,G_{11},G_{22}$ by means of the function $F\left(G,G_{11},G_{22}\right)$ minimization:

(76) $$F\left(G,G_{11},G_{22}\right)=\sum_{i}f^{2}\left(G,G_{11},G_{22},T_{i},x_{i}\right)=\text{min},$$

where $T_{i},x_{i}$ are the experimental points. As a result, system of equations for the model parameters $G,G_{11},G_{22}$ finding has the following form:

(77) $$\{\begin{array}{l} \frac{\partial F\left(G,G_{11},G_{22}\right)}{\partial G}=0;\\ \frac{\partial F\left(G,G_{11},G_{22}\right)}{\partial G_{11}}=0;\\ \frac{\partial F\left(G,G_{11},G_{22}\right)}{\partial G_{22}}=0. \end{array}$$

After parameters $G,G_{11},G_{22}$ finding it is possible to perform research of the Curie temperature as function of solution composition.

#### 3.2.8. System Ba_1−x_Pb_x_TiO_3_

There are the experimental data for Curie temperature of the system Ba_1−x_Pb**_x_**TiO_3_ at concentrations 0 ≤ *x* ≤ 1 [153,154,155,156,157]. The part of the measurement results presented in the Table 4. 

**Table 4:** Curie temperatures as a function of composition of Ba_1−x_Pb**_x_**TiO_3_.

x	0	0.5	1.0
T(x)	393 K	623 K	763 K

The first line contains the concentration of PbTiO_3_ in the system, the second line contains the corresponding Curie temperatures. Results of the parameters $G_{11}\;,\;G_{22}\;,\;G$ calculations are:

(78) $$\{\begin{array}{l} G_{11}=2358\text{K}\\ G_{22}=4578\text{K};\\ G=-4.043\cdot 10^{6}{\text{K}}^{2}. \end{array}$$

**Figure 6:**
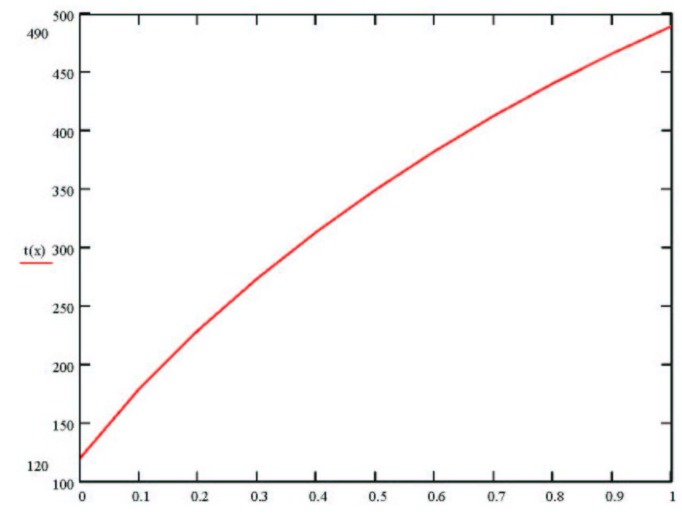
Calculated dependence of the Curie temperature of the system Ba_1-x_Pb_x_TiO_3_.

Results of these parameters using Curie temperatures of the solutions are presented in Figure 6. These results are in close agreement with experimental data.

Thus, the lattice model with effective field approximation for long-range parts of the interatomic potentials can be used for description of the ferroelectric solid solutions. But, the essential restriction of the model should be noted: this approach in the present form can be realized in the case of similarity of the crystal structures of the solid solution components only. Otherwise the method should be modified.

## 4. Modeling of Magnetoelectric Composites

The theory for 3-0 composites is based on the cubic model of composite [158,159]. The sample is assumed to consist of cubes. Dimensions of sample are supposed to be small compared with wave-lengths of AC fields used in the measurements. One, therefore, needs to obviously analyze only one of the units to describe the whole sample. For a unit cube, Equations (79) and (80) are described. The boundary conditions consist in force balance and equality of medium displacement on the boundaries. Using Equations (79) and (80) and boundary conditions enables one to obtain ME voltage coefficient numerically. Calculations show that the peak longitudinal ME voltage coefficient for 3-0 connectivity reaches 4000 mV/cm Oe and is three times higher than the transverse coefficient when the poling direction is perpendicular to the bias magnetic field. In case of 0-3 connectivity the peak longitudinal ME voltage coefficient equals 900 mV/cm Oe. In a real composite sample, the internal units are clamped by neighboring ones. Taking into account the clamping effect caused by surrounding unites cubes leads to significant decreasing of ME voltage coefficients. 

**Figure 7:**
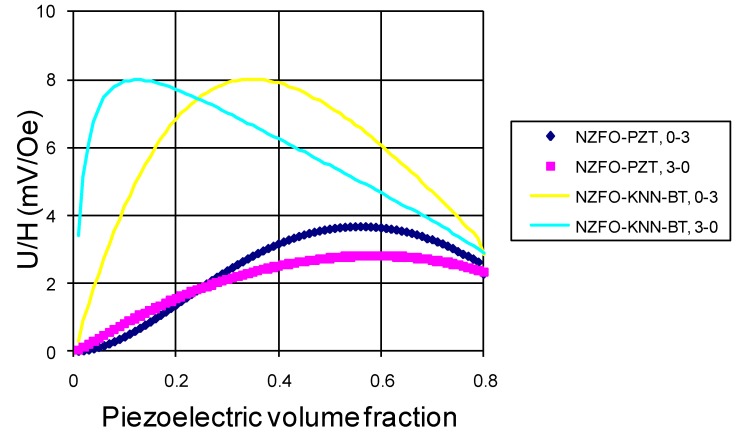
Piezoelectric volume fraction dependence of ME voltage coefficient for 3–0 and 0–3 composites of NZFO-PZT and NZFO-KNNBT.

PZT volume fraction dependence of transverse ME voltage coefficients is shown in Figure 7 for the sample in which internal units are assumed to be clamped by neighboring ones.

Further, we consider only symmetric extensional deformation in this model and ignore any asymmetric flexural deformations of the layers that would lead to a complex solving procedure. Our consideration is based on the following equations that can be written for the polarized piezoelectric phase with the symmetry ∞*m* for the strain and electric displacement:

*^p^S_i_ = ^p^s_ij _^p^T_j_ + ^p^d_ki _^p^E_k_* *^p^D_k_ = ^p^d_ki _^p^T_i_ + ^p^ε_kn _^p^E_n_* (79)

where *^p^S_i_* and *^p^T_j_* are strain and stress tensor components of the piezoelectric phase, *^p^E_k_* and *^p^D_k_* are the vector components of electric field and electric displacement, *^p^s_ij_* and *^p^d_ki _* are compliance and piezoelectric coefficients, and *^p^*ε*_kn_* is the permittivity matrix.

The magnetostrictive phase is assumed to have a cubic symmetry and is described by the equations:

*^m^S_i_ = ^m^s_ij _^m^T_j_+^m^q_ki_^m^H_k_* (80)

where *^m^S_i_* and *^m^T_j_* are strain and stress tensor components of the magnetostrictive phase, *^m^H_k_* and are the vector components of magnetic field, *^m^s_ij _* and *^m^q_ki_* are compliance and piezomagnetic coefficients.

The layers are assumed to be perfectly coupled at the interface and we use the following boundary conditions:

*v^ p^T_i_ + (1 − v)^m^T_i_ =* 0 and *^p^S_i_ = ^m^S_i_* for *i=*1, 2, (81)

where *v* is PZT volume fraction.

Equations (79)–(81) yield the expression for ME voltage coefficient for longitudinal fields’ orientation as:

(82) αE 33=E3H3=2μ0v(1−v)d31pq31m{2d312p(1−v)+ε33p[(s11p+s12p)(v−1)−v(s11m+s12m)]}×[(s11p+s12p)(v−1)−v(s11m+s12m)]{[μ0(v−1)−μ33mv][v(s12m+s11m)−(s12p+s12p)(v−1)]+2q312mv2}

where *E_k_* and *H_k_* are vector components of the electric and magnetic field; *s_ij_* is an compliance coefficient; *d_ki_* is a piezoelectric coefficient; *q_ki_* is a piezomagnetic coefficient; *ε_kn _* is permittivity.

For transverse fields’ orientation, the following expression can be obtained for ME voltage coefficient:

(83) αE,31=E3H1=−v(1−v)(q11m+q21m)d31pε33p(s12m+s11m)v+ε33p(s11p+s12p)(1−v)−2d312p(1−v)

As an example, numerical estimates are made for bilayers of NZFO, cobalt freite (CFO) or Ni and PZT or lead-free ferroelectrics. Using the zinc-substituted nickel ferrite is dictated by fact that when Zn is substituted in nickel ferrite, the room temperature *q* varies linearly with increasing Zn concentration x for x < 0.3 [160]. The material parameters used for theoretical estimates are listed in Table 5. Calculations of magnetically induced voltage are for the sample thickness of 1 mm.

**Table 5:** Material parameters (compliance coefficient s, piezomagnetic coupling q, piezoelectric coefficient d, and permittivity ε) for NZFO, CFO, Ni, PZT and lead-free ferroelectrics [161,162] used for theoretical estimates.

Material	s_11_ (10^−12 ^m^2^/N)	s_12_ (10^−12 ^m^2^/N)	q_11_ (10^−12 ^m/A)	q_12_ (10^−12 ^m/A)	d_31_ (10^−12 ^m/V)	d_33_ (10^−12 ^m/V)	ε_33_/ε_0_
PZT	17.3	−7.22	-	-	−175	400	1750
NBT-BT	7.3	−3.2	-	-	−140	280	2000
Mn:NBT-BT	7.3	−3.2			−242	483	4000
NKN-BT	5.55	−1.04			−110	225	1058
NZFO	6.5	−2.4	−1050	210			-
CFO	6.5	−2.4	−1880	556			
Ni	4.9	−1.5	−4140	570			

**Figure 8:**
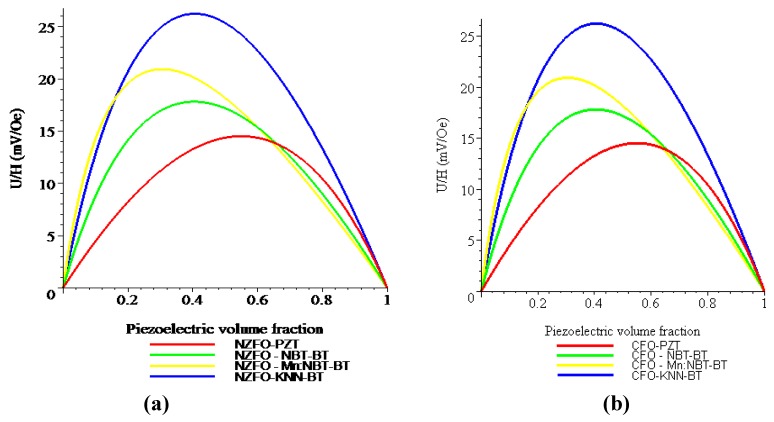
Piezoelectric volume fraction dependence of longitudinal magnetoelectric (ME) voltage coefficient for bilayers of NZFO and piezoelectrics **(a)** and CFO and piezoelectrics **(b)**.

Figure 8 shows the piezoelectric volume fraction dependence of ME voltage coefficient for NZFO-PZT, NZFO-MBT-BT, NZFO-Mn:MBT-BT, and NZFO-NKN-BT and for CFO-PZT, CFO-MBT-BT, CFO-Mn:MBT-BT, and CFO-NKN-BT for longitudinal fields’ orientation.

**Figure 9:**
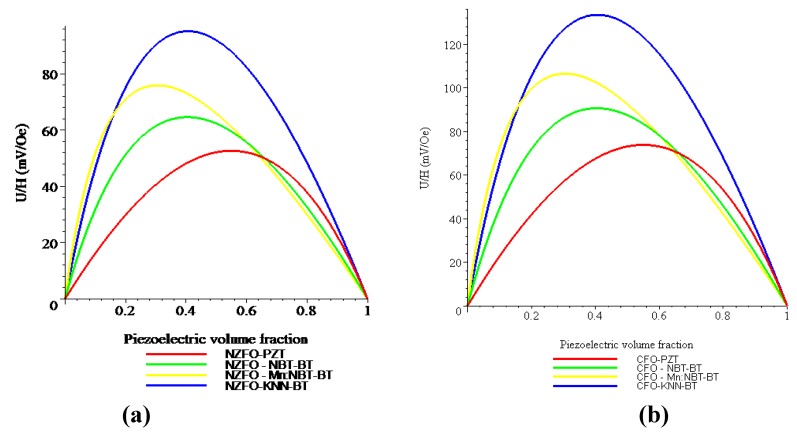
Piezoelectric volume fraction dependence of transverse ME voltage coefficient for bilayers of NZFO and piezoelectrics **(a)** and CFO and piezoelectrics **(b)**.

**Figure 10:**
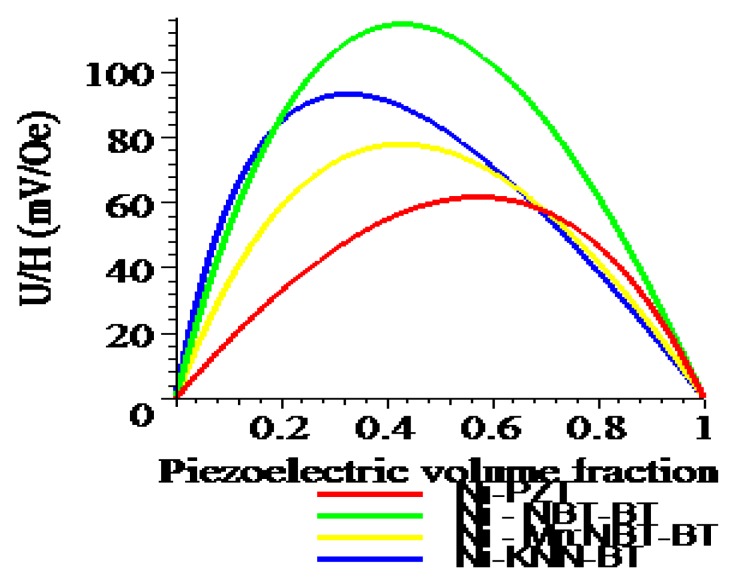
Piezoelectric volume fraction dependence of longitudinal ME voltage coefficient for a bilayer of Ni and piezoelectrics.

**Figure 11:**
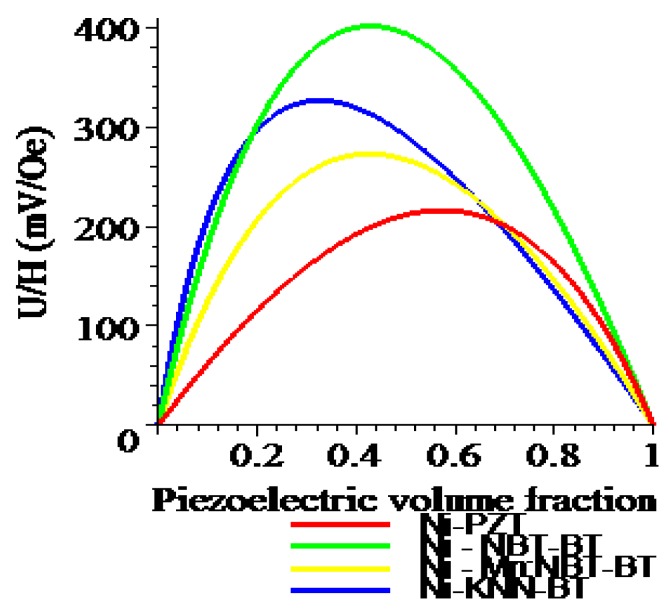
Piezoelectric volume fraction dependence of transverse ME voltage coefficient for a bilayer of Ni and piezoelectrics.

One can see from Figure 8 and Figure 9 that the transverse ME voltage coefficient considerably exceeds the longitudinal one that is attributed to a reduction in the internal magnetic field due to finite magnetic permeability of ferrite and demagnetizing fields for longitudinal fields’ orientation. The stronger piezomagnetic coupling for CFO (see Table 5) results in increased ME coefficients compared to NZFO. Figure 10 and Figure 11 show the futher increase of ME effect at the replacement of ferrites by a Ni phase.

**Figure 12:**
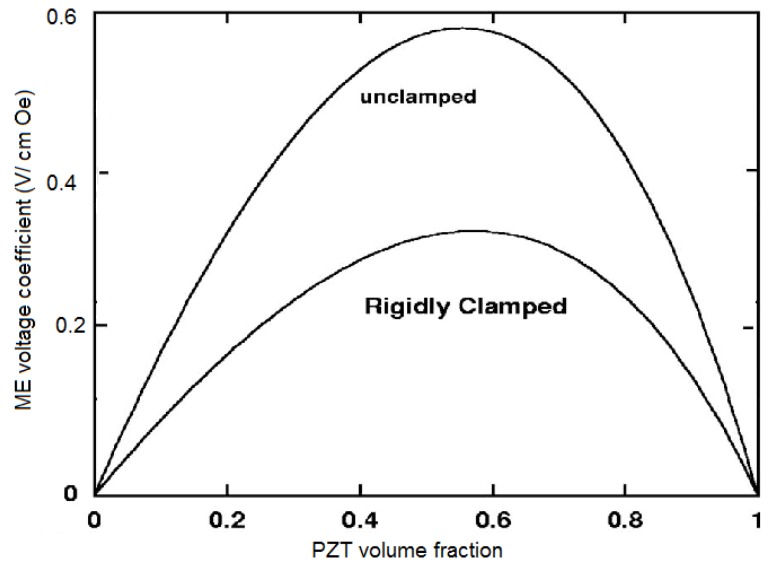
PZT volume fraction dependence of ME voltage coefficient for free and rigidly clamped bilayers of NZFO and PZT for transverse fields’ orientation.

The above theory is for samples that are free of any external mechanical force. Now we consider bilayers that are clamped perpendicular to the plane of the bilayer, along direction 3. The compliance of the clamp system is represented by *s_c__33_*, with zero compliance for rigidly clamped samples and infinite compliance for unclamped samples. As an example, we consider ME coupling in a clamped bilayer for transverse field orientation. The boundary conditions for the clamped sample become *T1 = 0*,* T2= 0*, and T3 = *s_c__33_ T_3_*. The transverse ME voltage coefficient is determined by the expression (84)

(84) $$\alpha_{E,31}=\frac{\alpha_{31}(s_{33}+s_{c33})-d_{33}q_{33}}{\varepsilon_{33}(s_{33}+s_{c33})-d_{33}^{2}}$$

where *α_33_*,* s_33_*,* d_33_, q_33_*, and *ε_33_* are ME susceptibility, effective compliance, piezoelectric and piezomagnetic coefficients, and permittivity of composite [8]. The numerical estimate of ME coupling is presented in Figure 12 for free and rigidly clamped bilayers of PZT and NZFO. One can see from Figure 12 that clamping the sample reduced ME coupling by half.

## 5. Data on Magnetoelectric Response in 3-0, 2-2, and 1-3 Composites

### 5.1. ME Effect in Sintered Composites

**Figure 13:**
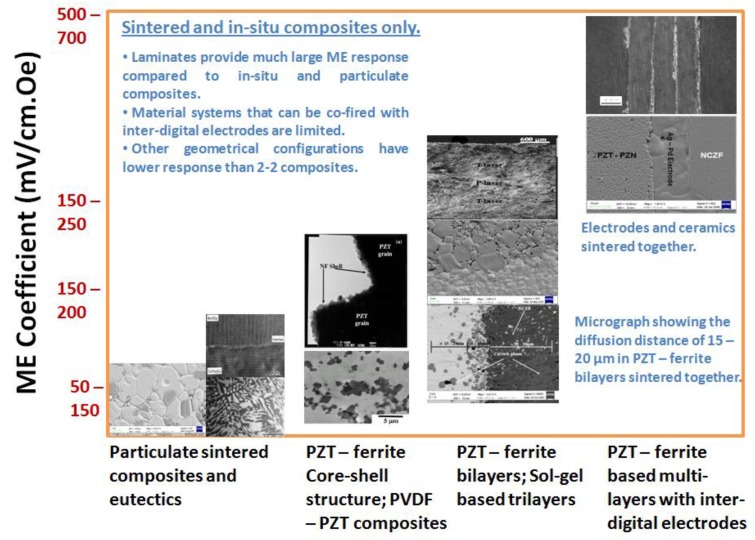
Magnetoelectric coefficient for various bulk sintered composites.

Magnetoelectric composite materials consisting of piezoelectric and magnetostrictive phases respond to both electric and magnetic field. The composites exploit product property and various synthesis techniques can be adopted to combine two different phases depending upon the crystal symmetry, lattice parameters and physical state. The presence of piezoelectric and magnetostrictive phases in the same material provides the opportunity to develop a voltage gain device operating on the following principle. An applied AC magnetic field induces strain in the magnetostrictive phase which is transferred onto the piezoelectric phase in an elastically coupled system. The piezoelectric phase produces the electric charge in proportion to the applied strain. Figure 13 summarizes the magnitude of ME coefficient reported for *in situ* and particulate sintered composites [12,24,163,164,165,166]. It can be immediately noticed that multi-layer laminates with inter-digital electrodes provide the highest magnitude of ME coefficient. Figure 13 also shows the interface microstructure in Pb(Zr,Ti)O_3_-(Ni,Zn)Fe_2_O_4_ (PZT-NZF) composites with bilayer geometry with no intermediate electrodes. By tuning the concentration of ionic dopants it is possible to achieve dense interfacial structures which can compensate for the lattice mismatch between the perovskite and spinel. However, diffusion of Zn and Cu ions into piezoelectric phase and Pb ions into magnetostrictive phase limits the magnitude of maximum achievable ME coupling. The diffusion length in Figure 13 is of the order of 15–20 μm with synthesis temperatures in the range of 1100 °C. In particulate composites there is additional problem limiting the magnitude of ME coupling, namely 3D connectivity of magnetic phase which for smaller piezoelectric grain sizes can be noticeable. It is now well understood that ME coefficient in particulate composites can be tailored to their maximum magnitude by synthesizing high piezoelectric voltage coefficient “g” and high piezoelectric strain coefficient “d” phase surrounded by low magnetic coercivity and high magnetization magnetostrictive phase. Additional constraint is imposed by the equivalence of mechanical impedance.

**Figure 14:**
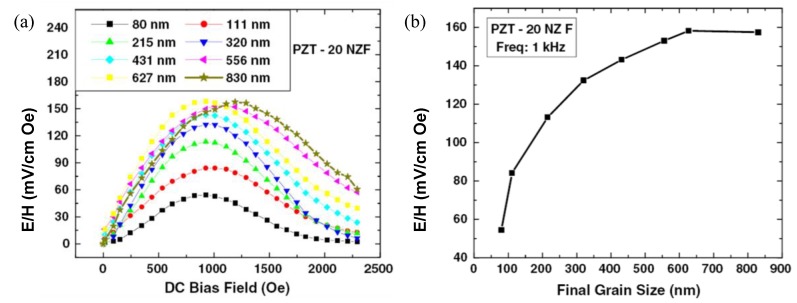
Magnetoelectric responses as a function of **(a)** DC bias field and **(b)** grain size of PZT.

Figure 14(a) and (b) shows the variations in ME coupling with varying piezoelectric grain size in 3–0 particulate Pb(Zr_0.52_Ti_0.48_)O_3_ (PZT)-Ni_0.8_Zn_0.2_Fe_2_O_4_ (NZF) composites [167]. It was found that as the piezoelectric phase grain size increases the overall resistivity, piezoelectric, dielectric and ferroelectric property of the composite increases and saturates above 600 nm. Below 200 nm average grain size, piezoelectric and dielectric properties decrease rapidly. The size effect of magnetic phase was small. In addition to diffusion, the main problem limiting the magnitude of ME coupling was found to be 3D connectivity of the magnetic phase, which for smaller piezoelectric grain sizes can be noticeable.

In layered composites the connectivity is limited to the region near the interface. For thin film systems, this may form the significant fraction of the overall system. Self-assembled microstructures (such as eutectic decomposition and strain minimization) suffer from the drawback that ferroelectric polarization is reduced in the system due to formation of mechanical twins, thus exhibiting low magnitude of ME coupling. BaTiO_3_-CoFe_2_O_4_ (BTO-CFO) system particulate composite with eutectic composition were reported by Echigoya *et al*. [163]. Even though the phase distribution occurs with high periodicity and interface exhibits crystallographic orientation relationships, the magnitude of ME coupling remains low. The orientation relationships between phases in eutectic decomposition of BTO-CFO can be expressed as: (i) for hcp BaTiO_3_: (111)CFO//(00.1)BTO and (110)CFO//(11.0)BTO; and (ii) for tetra/cubic BaTiO_3_: (001)CFO//(001)BTO and (100)CFO//(100) BTO. 

**Figure 15:**
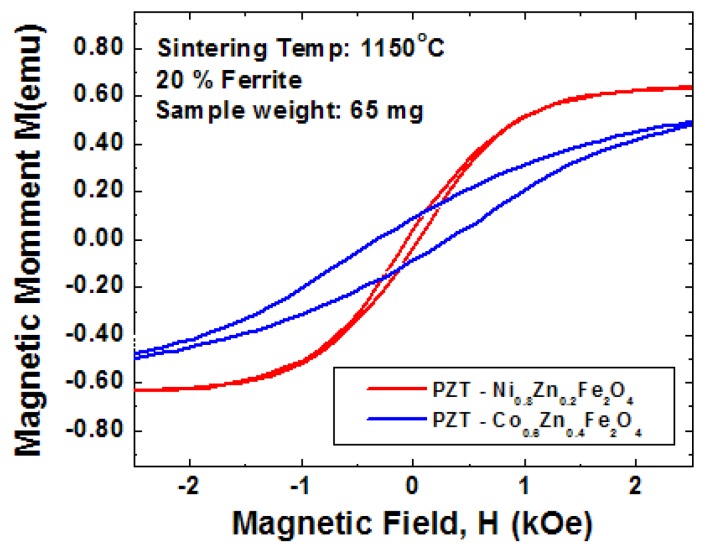
Comparison of magnetic properties for PZT-NZF and PZT-CZF.

High magnetostrictive coefficients are obtained in the compounds of the type R–T where R is rare earth and T is the transition metal, however these materials have poor resistivity and are chemically reactive. Thus, the choice for the magnetostrictive phase in grown / sintered composites narrows down to the spinel ferrites. In the spinel ferrites, the spontaneous magnetization corresponds to the difference between the sublattice magnetizations associated with the octahedral and tetrahedral sites. Results have shown enhanced magnitude of the ME coefficient for Ni_0.8_Zn_0.2_Fe_2_O_4_ (NZF) and Co_0.6_Zn_0.4_Fe_2_O_4 _(CZF). In the nickel zinc ferrite solid solution (Ni_1−x_Zn_x_Fe_2_O_4_) as x is increased Zn^2+^ replaces Fe^3+^ in the tetrahedral sites and Fe^3+^ fills the octahedral sites emptied by Ni^2+^. The net magnetization of nickel zinc ferrite is proportional to 5(1 + x) + 2(1 − x) − 0(x) − 5(1 − x) = 2 + 8x. Thus, the magnetic moment as a function of the Zn content increases until there are so few Fe^3+^ ions remaining in tetrahedral sites that the superexchange coupling between tetrahedral and octahedral sites breaks down. Figure 15 shows our results on the PZT-NZF and PZT-CZF composites. It can be seen from this figure that CZF is a hard magnetic phase, requires higher DC bias, has lower remanent magnetization and results in larger reduction of the ferroelectric polarization as compared to NZF. On the other hand, a high increase in the resistivity of the Ni-ferrites is obtained by doping with Co. Further, the sintering of Ni-ferrites has been found to be simpler with the PZT matrix due to low temperatures and adequate grain growth. 

**Figure 16:**
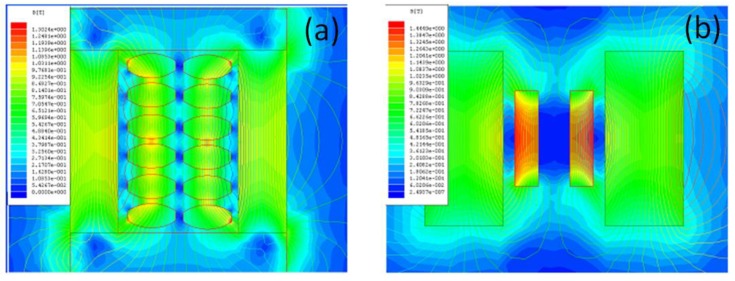
Magnetic field patterns **(a)** when the ferrite particles are placed between the magnets; and **(b)** when ferrite plates are placed.

In particulate-sintered composites consisting of random distribution of magnetostrictive particles, there is excessive cross-diffusion of ions across the interface. Recently, it was shown in Cu-modified nickel zinc ferrite (NCZF)–PZNT composites that Cu ions diffuse into PZNT while Pb ions diffuse into NCZF. This cross-diffusion lowers the magnitude of magnetostrictive constant and piezoelectric voltage constant. Another drawback of particulate-sintered composites is connectivity of the ferrite particles which lowers the overall resistivity and reduces the poling voltage. However, by confining the distribution of NFO along the grain boundaries and controlling the fraction of such boundaries, higher poling voltage can be applied. Figure 16(a) and (b) shows the simulation of the magnetic field pattern when the ferrite particles and ferrite plates are placed in between the strong DC magnetic field. The magnetic field pattern clearly reveals that the plates are able to reach the saturation state for a given applied magnetic field while dispersed particle do not because the flux line originating from the particles shield the response. This data provides another explanation for the experimentally observed fact that magnetoelectric response of the sintered *in situ* composites is lower than the layered ones. The field in textured nanocomposite can be enhanced by coupling with the external layers. 

**Figure 17:**
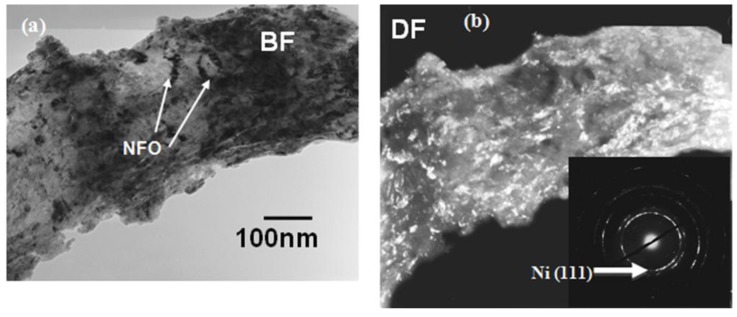
Transmission electron microscope images of PZT-NFO core-shell composite synthesized by high pressure compaction sintering: **(a)** Bright field **(b)** Dark field.

Recent results by Grossinger *et al*. on composite consisting of 50% cobalt ferrite-50% barium titanate in core-shell structure show giant increase in ME coefficient as compared to randomly dispersed composites [168]. The results were analyzed in terms of coupling coefficient k, given as k_par_ = λ_par_dλ_par_/dH, where λ_par_ represents the longitudinal magnetostriction, H is the applied magnetic field, and d is the effective sample thickness. It was shown that the magnitude of ME coefficient increases with k which is higher for core-shell structure. These results are consistent with our own data which shows that core-shell Pb(Zr,Ti)O_3_ (PZT)-NiFe_2_O_4_ (NFO) particulate nanocomposites provide higher magnitude of ME coefficient. In our case, core-shell composites were synthesized through high pressure sintering which resulted in good interface bonding and effective strain transfer across the interface [169]. The microstructure in Figure 17, which has a resemblance to core-shell structure, can provide effective elastic coupling between the magnetostrictive and piezoelectric phases. The magnitude of magnetostriction coefficient, λ_par_, and k will be higher for the NFO-ordered phase distribution along the grain boundaries due to texturing and phase separation.

**Figure 18:**
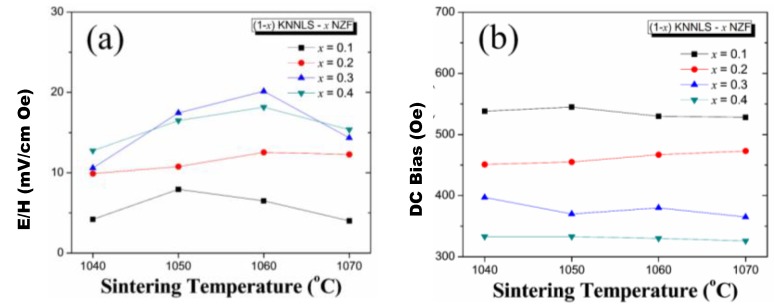
**(a)** ME coefficient and **(b)** *H_bias_* for (1 − x) KNNLS–x NZF composites.

The lead-free 3-0 ME composites have been studied using three types of piezoelectric materials based on: (i) K_0.5_Na_0.5_NbO_3_, (ii) Na_0.5_Bi_0.5_TiO_3_, and (iii) BaTiO_3_. BaTiO_3_-CoFeO_4_ (BTO-CFO) lead-free composites were reported by Boomgaard *et al*. in 1978 and were found to exhibit ME coefficient of 130 mV/cm∙Oe [13]. After that, several studies have been conducted on lead-free ME composites but the magnitude of ME coefficient was found to be below 100 mV/cm∙Oe in off-resonance conditions because of the poor piezoelectric properties of lead-free materials. Recently, lead-free 3-0 ME composites of (1 − x) [0.948 K_0.5_Na_0.5_NbO_3_–0.052 LiSbO_3_] − x Ni_0.8_Zn_0.2_Fe_2_O_4_ (KNNLS-NZF) with island–matrix structure have been studied by Yang *et al*. [170]. Figure 18 shows that the ME voltage coefficient was found to maximize in the 0.7 KNNLS–0.3 NZF sintered at 1060 °C and the optimum DC bias representing the maximum ME voltage coefficient decreased with increase in mole fraction of NZF in the composites. From the result in Figure 18(a), it can be also seen that the island-matrix system is a promising structure to use wide range of magnetic values with high ME effect.

### 5.2. ME Effect in 2-2 Composites

**Figure 19:**
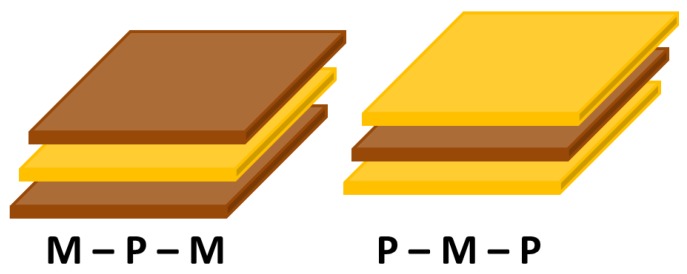
Schematic diagram of ME trilayer composites with two-type configurations.

A 2-2 connectivity refers to a bilayer consisting of piezoelectric and magnetostrictive phases [8]. The leakage problem due to high concentration of ferrite and low resistivity in the ferroelectric ceramic matrix can be eliminated through laminate structure as shown in Figure 19. However, high temperature co-firing process for the piezoelectric and ferrite ceramic layers is a big challenge due to difference in shrinkage rate, thermal expansion mismatch, and interdiffusion and/or chemical reactions between the two ceramic layers during the sintering process at high temperature. These composites also exhibit much larger anisotropy as compared to particulate composites.

**Figure 20:**
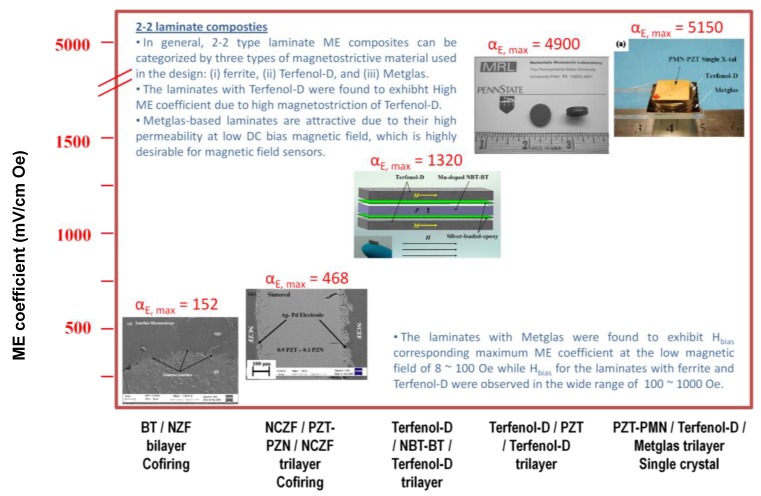
Magnetoelectric coefficient for 2-2 laminate composites in off-resonance conditions.

Figure 20 summarizes the magnitude of ME coefficient reported for 2-2 laminate composites in off-resonance conditions. In general, 2-2 laminate ME composites, three types of magnetostrictive mateirals have been used: (i) ferrite, (ii) Terfenol-D, and (iii) Metglas. Various ferrite oxides in Table 6 are available to co-sinter with piezoelectric matrix and were found to exhibit different magnetostrictions due to their varying magnetic properties [171]. Terfenol-D has the largest magnetostriction of 1400 × 10^−6^ with low permeability, while Metglas has extremely high relative permeability of >40,000 and thus is an quite attractive magnetostrictive material in low field ME sensors [172]. Detailed research has been performed by the authors exploring the 2-2 connectivity of the ME composites in the form of bilayers, trilayers and multilayers of piezoelectric and magnetostrictive materials. Islam *et al*. have synthesized PZT-NCZF bilayer and NCZF-PZT-NCZF trilayer using co-firing technique. We found a high ME coefficient on the order of ~500 mV/cm·Oe in trilayer structures [164]. Lead-free 2-2 laminate ME composites of BaTiO_3_ (BT) / (Ni_0.8_Zn_0.2_)Fe_2_O_4_ (NZFO) also were synthesized by co-firing and the magnitude of ME voltage coefficient reached saturation at DC bias of 1000 Oe with maximum magnitude of 152 mV/cm Oe [173]. Dong *et al*. have studied several multilayered ME configurations by using materials PZT, Terfenol D and Metglas and have reported quite high ME coefficients on the order of 10 V/cm·Oe in resonance condition [30,32,33,38]. Among bulk multiferroic composites, the composites consisting of giant magnetostrictive alloy Tb_1–x_Dy_x_Fe_2_ (Terfenol-D) and piezoelectric Pb(Zr,Ti)O_3_ (PZT), exhibiting high magneto and electromechanical energy densities respectively, are the most attractive due to their giant ME response [33,34,38,174,175]. Recent research by Park *et al*. reported the maximum ME coefficient of 5150 mV/cm∙Oe in off-resonance condition by combining PZT-PMN, Metglas, and Terfenol-D [176].

**Table 6:** Magnetostrictions for ferrite oxides.

Material	$\lambda s(\times \;10^{-6})$
MnFe_2_O_4_	−5
Fe_3_O_4_	40
CoFe_2_O_4_	−110
MgFe_2_O_4_	−6
Li_0.5_Fe_2.5_O_4_	−8
NiFe_2_O_4_	−26
CuFe_2_O_4_	−9
YFe_5_O_12_	−2
SmFe_5_O_12_	3.3
DyFe_5_O_12_	1.46
EuFe_5_O_12_	9.48

**Figure 21:**
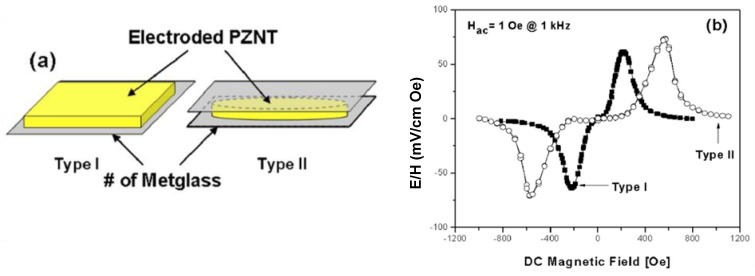
**(a)** Schematic diagram and **(b)** ME voltage coefficients for PZNT/Metglas bilayer and Metglas/PZNT/Metglas trilayer laminates.

Our study on texturing in 2-2 laminate composites shows that the cofired ME trilayer consisting of (Ni_0.6_Cu_0.2_Zn_0.2_)Fe_2_O_3 _(NCZF) and 0.85Pb(Zr_0.52_Ti_0.48_)O_3_-0.15Pb(Zn_1/3_Nb_2/3_)O_3_ (PZT–PZN) with partial texturing was found to exhibit 67 % improvement in magnitude of ME coefficient than that of trilayer with random orientation [177]. The textured PZT-PZN composites with high tetragonality were found to possess 44% improvement in *d_33_* and 44–50% enhancement in dielectric constant. Park *et al*. reported ME responses in 2-2 laminate composites consisting of Pb(Zn_1/3_Nb_2/3_)_x_(Zr_0.5_Ti_0.5_)_1−x_O_3_ (PZNT) and Metglas [178]. These laminates were fabricated by stacking 20 layers of Metglas on one-side of PZNT plate for type I and both-side of PZNT plate for type II composites as shown in Figure 21. The ME voltage coefficients were found to be 62 mV/cm Oe at DC bias of 215 Oe for type I and 73 mV/cm Oe at DC bias of 570 Oe. Dimensionally gradient bimorph structure has been designed for wide operating range of frequency and *H_bias_* [179]. The ME behavior was found to be dependent on both shape and dimension of laminates such that the bimorph laminates with asymmetric H shape were found to exhibit flat ME responses by merging different dimensional ME responses under wide ranges of *H_bias_* = 60−215 Oe and *f* = 7−22 kHz, respectively. Our results have laid the foundation for design of the magnetic field sensors exhibiting wide frequency and DC bias operating range.

**Figure 22:**
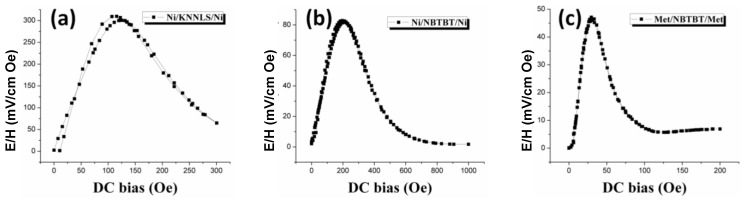
ME voltage coefficients for 2-2 trilayer laminates of **(a)** Ni/NKNLS/Ni; **(b)** Ni/NBTBT/Ni; and **(c)** Metglas/NKNLS/Metglas.

Recently, we have measured the ME voltage coefficients for 2-2 lead-free ME laminates given as Ni/NKNLS / Ni, Ni/NBTBT/Ni, and Metglas/NBTBT/Metglas. These lead-free ME laminates were fabricated by embedding piezoelectric layers between magnetostrictive layers of Ni plates or Metglas. The lead-free laminates with Ni in Figure 22 (a) and (b) were found to exhibit high ME coefficients of 300 and 80 mV/cm·Oe at *H_bias_* = 100 and 200 Oe, respectively. On the other hand, the laminates with Metglas shown in Figure 22 (c) show the maximum ME coefficient of 47 mV/cm·Oe below 30 Oe due to the high permeability of Metglas.

**Figure 23:**
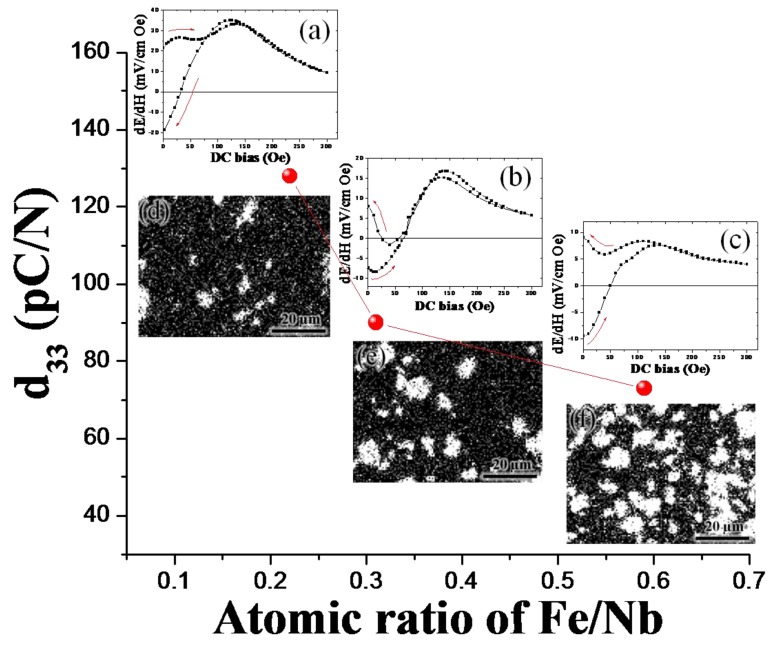
Longitudinal piezoelectric strain coefficient for (1 − x) KNNLS–x NZF composites as a function of atomic ratio of Fe/Nb. ME coefficient for (1 − x)KNNLS-xNZF / Ni / (1 − x)KNNLS-xNZF bending mode laminates: **(a)** x = 0.1; **(b)** x = 0.2; and **(c)** x = 0.3. Fe (white color) mapping images for (1 − x)KNNLS-xNZF composites: **(d)** x = 0.1; **(e)** x = 0.2; and **(f)** x = 0.3.

Effect of loss factors on dynamic ME response in 2-2 laminates has been reported by Cho *et al*. [180]. It was shown that the magnitude of ME sensitivity is dependent on intensive dielectric and piezoelectric loss in sub-resonance conditions but dominantly dependent on extensive mechanical loss in resonance conditions. We have designed ME laminate composites given as Pb(Zr,Ti)O_3_-Pb(Mg_1/3_Nb_2/3_)O_3_ single crystal/Terfenol-D/Metglas for high sensitivity and found ME coefficient of 5 V/cm Oe at 1 kHz with DC magnetic sensitivity of 500 nT and 1 μT [176]. Recently, we have reported self-biased ME response, which is defined as remnant ME coefficient at zero *H* bias, in 2-2 ME laminate composites consisting of both one-phase piezoelectric and two-phase magnetostrictive layers with electric connection for bending [181]. The ME hysteresis was found to be dependent on both magnetic interaction and bending effect, and various shapes of ME hysteresis could be obtained in off-resonance and resonance conditions. Recently, the material and structural effects on self-biased ME responses was realized by changing material composition, so that one could control the shape of ME hysteresis which can be promising for applications in low-field/high sensitive magnetic sensors and electrically, tuned memory devices as shown in Figure 23 [170].

### 5.3. ME Effect in 1-3 Composites

**Figure 24:**
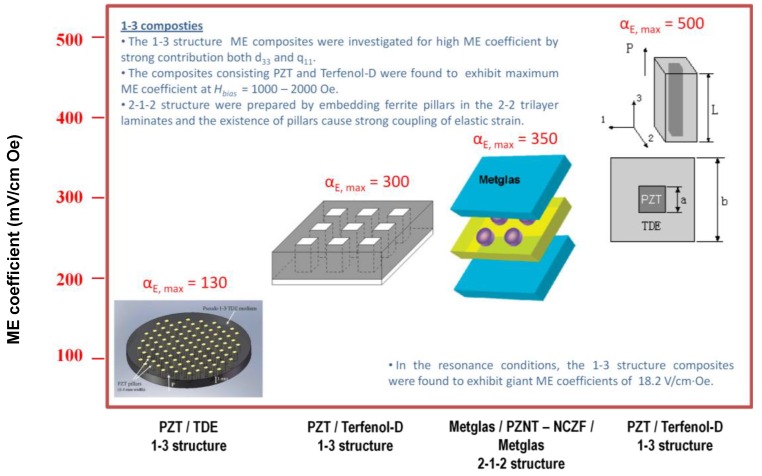
Magnetoelectric coefficient for 1-3 composites in off-resonance conditions.

The ME coupling in 1-3 structure is considerably stronger than in 2-2 structure. In fact, ME effect in a 2-2 composite is determined by piezoelectric coefficient *d_31_* and piezomagnetic coefficient *q_11_ + q_12_*. For a 1-3 structure, the determining contribution arises from *d_33_* and* q_11_* since the stress components along the cylinders axis substantially exceed other components. Piezoelectric and piezomagnetic coefficients *d_33_* and* q_11_* are known to be about two times greater compared than *d_31_* and *q_11_ + q_12_*. In case of 3-0 composite, the observed decrease in ME coupling strength is caused by clamping the internal units of cubic model by neighboring ones. Clamping restricts the strains of composite and reduces the induced voltage across the piezoelectric phase. Figure 24 summarizes the magnitude of ME coefficient reported for 1-3 composites in off-resonance conditions. 

**Figure 25:**
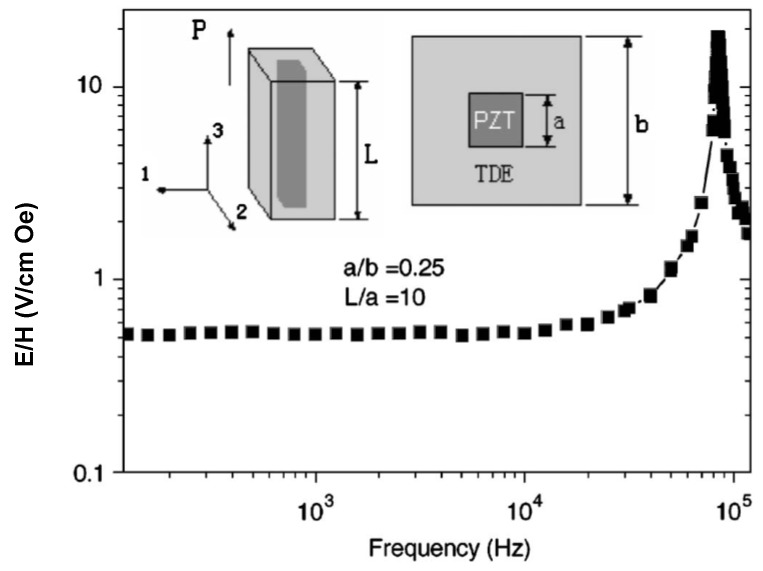
Frequency dependence of the ME coefficients for the small ME composite rod with a/b = 0.25 and L/a = 1 mm.

Several reports have been published in literature in order to achieve magnetic and magnetoelectric nanowires/nanocomposites exhibiting 1–3 mode. The development of the soft mold process allows for the preparation of fine scale 1–3 composites with PZT rods of different size, shape and spacing, which can be used as ultrasonic transducers for frequencies 55 MHz [182]. Ma *et al*. showed a single period of 1-3-type structured ME composite as shown in Figure 25. The experimental results demonstrated that the coupling interaction between the PZT rod and TDE medium can generate much larger ME response. Especially at high frequency where the electromechanical resonance appears, the composite shows a giant ME effect. This single period 1-3-type composite presents a size-dependent ME response, Figure 24 indicates that micro/nano ME pillars with large ME response can be obtained and promises future micro-ME devices. Lam *et al*. reported frequency response of 1-3 ME composites consisting of Pb(Zr,Ti)O_3_ (PZT) rods embedded in a matrix of Terfenol-D/epoxy (TDE) [183]. The composites were found to exhibit maximum ME coefficient of 130 mV/cm·Oe at 1 kHz and the resonance shifts to lower frequency with increase in bias field. Also, the pseudo 1-3 ME composite consisting of PZT rod array and Terfenol-D/epoxy matrix were reported by Shi *et al*. [184] and the magnetoelectric coefficients were found to be 300 mV/cm·Oe at off-resonance frequency and 4500 mV/cm·Oe at resonant frequency.

We consider a structure consisting of piezoelectric and magnetostrictive coaxial cylinders. Both cylinders are assumed to be ideally bonded together. Such a system is an example of 1-3 composite. For the sake of simplicity, we consider a poled state in both the piezoelectric and magnetostrictive phases with the poling directed along the axis of cylinders (*z* axis). The applied ac magnetic field is also assumed to be directed along *z* axis. As a result, the induced electric field also has the same direction. How to solve the problem of ME coupling in a 3-1 or 1-3 magnetostrictive-piezoelectric nanocomposite on a substrate is described in our previous work [185]. To adapt that model to the structure to be considered, we ignore the substrate clamping and lattice mismatch effect. Taking into account the axial symmetry of structure, the elastostatic equation is written in cylindrical coordinates system:

(85) $$\frac{\partial T_{rr}}{\partial r}+\frac{1}{r}(T_{rr}-T_{\theta \theta })=0.$$

Transferring Equations (79) and (80) to cylindrical coordinates and expressing the stress tensor component in terms of strain tensor component, one can substitute the found expressions into Equation (85). As a result, the following equation can be obtained for the radial displacement* u_r_* that defines strain components *S_rr_=∂u_r_/∂r* and *S_θθ_=u_r_/r* for both phases:

(86) $$\frac{\partial^{2}u_{r}}{\partial r^{2}}+\frac{1}{r}\frac{\partial u_{r}}{\partial r}-\frac{u_{r}}{r^{2}}=0.$$

The boundary conditions in this case have the form:

*^p^u_r_ =0* at* r=0,* *^p^u_r_ = ^m^u_r_* and *^p^T_r_ = ^m^T_r_* at *r = ^p^R*

*^m^T_r_ = 0* at* r = ^m^R* (87)

*^p^S_zz_ = ^m^S_zz,_*

**Figure 26:**
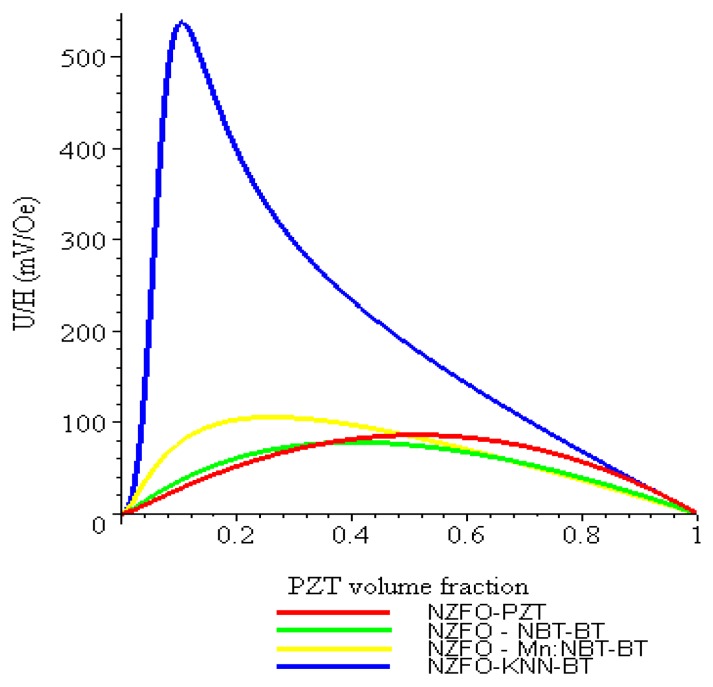
Piezoelectric volume fraction dependence of ME voltage coefficient for 1–3 composites of NZFO-PZT, NZFO-NBTBT, NZFO-Mn:NBTBT, and NZFO-NKNBT.

∫0RpTzzprdr= −∫RpRmTzzmrdr

where *^p^R* and *^m^R* are radii of piezoelectric and piezomagnetic phases. Equations (79), (86), (87) and open circuit condition D_3_ = 0 constitute a closed system and can be numerically solved for ME voltage coefficient *α_E,33_*. Estimates are shown in Figure 26 for NZFO-PZT, NZFO-MBT-BT, NZFO-Mn:MBT-BT, and NZFO-NKN-BT structures with sample thickness of 1 mm. It should be noted that the PZT volume fraction dependence of ME voltage coefficient for a 3–1 composite is similar to that of 1-3 composite in Figure 24.

**Figure 27:**
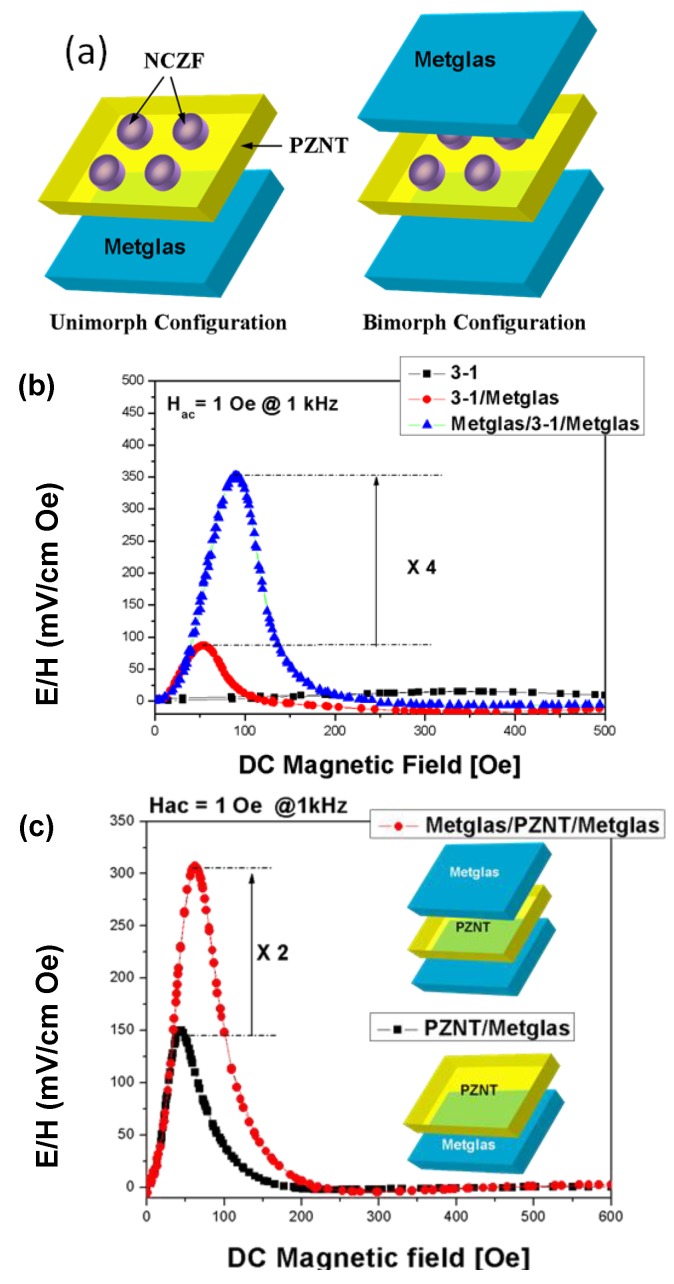
**(a)** Schematic diagrams of unimorph and bimorph configurations; **(b)** ME coefficient of 3-1 composite depending on the laminations: 3-1/Metglas and Metglas/3-1/Metglas; **(c)** ME coefficient of typical 2-2 composites based on PZNT and Metglas: unimorph and bimorph configurations.

In our study on 2-1-2 ME laminate composites having configuration of Metglas/0.2Pb(Zn_1/3_Nb_2/3_)O_3_-0.8Pb(Zr_0.5_Ti_0.5_)O_3_(PZNT)/Metglas with ferrite pillars embedded in the PZNT phase [186]. The piezoelectric layer with composition PZNT consisted of co-fired (Ni_0.6_Cu_0.2_Zn_0.2_)Fe_2_O_3_ (NCZF) pillars as shown in Figure 27 (a). The 2-1-2 composite was found to exhibit the ME coefficient of 352 mV/cm·Oe which is 15% higher magnitude than that for 2-2 laminate composites by effective coupling in the 2-1-2 structure as shown in Figure 27 (b) and (c). The result shows the existence of NCNF pillars in 2-1-2 laminates causes strong coupling of elastic strain.

## 6. Conclusions

In this manuscript, we report the ME characterization of composites based on magnetostrictive NZFO or Ni and piezoelectric PZT and lead-free ferroelectrics. The piezoelectric volume fraction dependence of ME coefficients is calculated using the physical parameters of composite components. In order to overcome the problem of toxicity of Pb, we conducted experiments with Pb-free piezoelectric compositions illustrating their importance as an environment friendly alternative. To estimate the piezoelectric parameters of lead-free materials, we used the thermodynamic LGD approach and generalized lattice model of ferroelectric solid solutions. It has been shown that composites based on lead-free ferroelectrics can exhibit the ME couling that is at least not weaker compared to leaded materials.
